# Macrophage Infiltration and ITGB2 Expression in ESCC: A Novel Correlation

**DOI:** 10.1002/cam4.70604

**Published:** 2025-01-17

**Authors:** Tao Huang, Longqian Wei, Huafu Zhou, Jun Liu

**Affiliations:** ^1^ Department of Cardiothoracic Surgery The First Affiliated Hospital of Guangxi Medical University Nanning People's Republic of China

**Keywords:** esophageal squamous cell carcinoma, immunotherapy, ITGB2, macrophages

## Abstract

**Background:**

Esophageal squamous cell carcinoma (ESCC) is one of the most prevalent and lethal malignancies worldwide. Despite progress in immunotherapy for cancer treatment, its application and efficacy in ESCC remain limited. Therefore, there is an ongoing need to explore potential molecules and therapeutic strategies related to tumor immunity in ESCC.

**Methods:**

In this study, we integrated high‐throughput sequencing data, gene chip data, single‐cell sequencing data, and various bioinformatics analysis methods along with experimental approaches to identify key genes involved in immune infiltration in ESCC and investigate their relationship with immune cell development, as well as the potential of these key genes in immunotherapy.

**Results:**

We discovered and validated a positive correlation between macrophage infiltration and ITGB2 expression in ESCC. ITGB2 is overexpressed in ESCC and has potential as a prognostic biomarker for the disease. We present for the first time the finding that the expression of ITGB2 in infiltrating macrophages increases as these macrophages polarize toward a tumor‐promoting phenotype in ESCC. Moreover, during the progression of ESCC, ITGB2 expression in infiltrating macrophages is upregulated. The higher the expression of ITGB2, the more feasible it is to target macrophages. Additionally, we found that evaluating immune therapy responses in ESCC patients through ITGB2 expression is a viable approach. Furthermore, we identified three miRNAs associated with abnormal ITGB2 expression, providing insights into the upstream molecular interactions of ITGB2.

**Conclusions:**

Macrophage infiltration in ESCC is closely associated with ITGB2, which holds significant potential for immunotherapy applications in ESCC. Based on our findings and prior studies, we propose a novel hypothesis: inducing M1 macrophages in vitro, knocking out ITGB2, and then reinfusing these ITGB2‐knockout M1 macrophages into ESCC patients may represent a promising new immunotherapy strategy, providing a new avenue for ESCC immunotherapy.

## 1 Introduction

The incidence and mortality rates of esophageal cancer remain high and are on the rise [1]. Squamous cell carcinoma, as its most common histological subtype, accounts for over 70% of all global esophageal cancer cases. Currently, the 5‐year survival rate for patients with esophageal squamous cell carcinoma (ESCC) is only around 20% [2]. Therefore, it remains a significant public health issue.

The primary treatment strategy for ESCC currently relies on comprehensive treatment, with surgery as the main approach. However, the overall treatment outcomes are not satisfactory [3]. With continuous advancements in tumor research, immunotherapy, as a novel treatment modality, has opened new avenues for ESCC treatment, following the success achieved in tumors such as lung cancer, renal cancer, and melanoma [4]. Some clinical trials have also achieved certain results, such as Checkmate‐577 [5], Keynote‐97 [6], and Keynote‐590 [7].

But, several factors hinder the further application of immunotherapy in ESCC. Studies have reported that patients initially responsive to immunotherapy tend to develop acquired resistance (AR) after receiving two or more courses of treatment [8]. Additionally, most current therapies require Programmed Cell Death 1 Ligand 1 (PDL1) positivity. As we all know, the fundamental principle [9] behind the use of PD1/PD‐L1 inhibitors in cancer treatment is that tumor cells evade immune surveillance by overexpressing PD‐L1, which then binds to PD1 on cytotoxic T cells, leading these T cells to recognize tumor cells as “normal.” By blocking the interaction between PD1 on cytotoxic T cells and PD‐L1 on tumor cells, PD1/PD‐L1 inhibitors enable cytotoxic T cells to continue recognizing and killing tumor cells. However, due to tumor heterogeneity, some patients present with PD‐L1‐negative tumors, where tumor cells evade immune cell attack through alternative mechanisms, rendering them unable to benefit from current treatment options [10]. Additionally, the tumor microenvironment (TME) is complex, comprising various immune cells whose infiltration also influences tumor progression and patient prognosis [4]. Thus, PD1/PDL1 is just a starting point, and further exploration of more tumor immune‐related biomarkers, targets, and molecular mechanisms is still needed in the future.

Today, with the development and maturation of high‐throughput sequencing, gene chips, single‐cell sequencing, and related bioinformatics analysis technologies, we have been provided with a wealth of tumor‐related genomic information, offering unprecedented opportunities to explore biomarkers, targets, and molecular mechanisms related to tumor immunity.

In this study, we will comprehensively employ high‐throughput sequencing, gene chips, single‐cell sequencing, and various bioinformatics analysis methods to explore pivotal genes involved in ESCC immune infiltration. We will delve into the relationship between these pivotal genes and the development of immune cells, as well as its potential applications in immunotherapy.

## 2 Materials and Methods

### 2.1 Data Source and Preprocessing

The gene chip data were obtained from four series in the GEO (Gene Expression Omnibus) database: GSE161533, GSE23400, GSE66274, and GSE67268. To enhance data reliability, all chip data underwent cleaning, standardization, inspection, and batch effect removal. Subsequently, probe annotation and gene ID conversion were performed.

The high‐throughput sequencing data were sourced from The Cancer Genome Atlas (TCGA) and the Genotype‐Tissue Expression (GTEx) Database. To ensure data reliability, we utilized the TCGA original count data from UCSC Xena and systematically checked each TCGA sample using the “Merged Sample Quality Annotations” file from the TCGA website, removing samples with poor quality. Only high‐quality samples with stage “A” were retained. Moreover, for ESCC samples, we selected samples with “Sample Type Codes” of “01”, “02”, “05”, or “08”. For normal tissue samples, we only chose samples with “Sample Type Codes” of “11”. Considering the different sources of data from TCGA and GTEx, direct merging analysis could introduce significant bias and affect the accuracy of results. Therefore, we utilized the “UCSC Toil RNAseq Recompute Compendium” data [11] from UCSC Xena for merged analysis to minimize bias arising from different data sources. For the original count data, batch effect correction was performed using the “batch_number” provided by UCSC Xena. Since the data from “UCSC Toil RNA‐seq Recompute Compendium” did not provide batch effect reference, we explored and removed batch effects using the R package “RUVSeq(v1.32.0)”.

The single‐cell sequencing data were obtained from the GEO database under accession number GSE199619. We utilized the “GSE199619_ELN.integrated.rds.gz” file. To minimize bias, we selected ESCC samples that underwent surgical resection or endoscopic resection without neoadjuvant chemotherapy (NACT) (Table S2).

Summary information for all data is provided in Table S1.

### 2.2 Immunoinfiltration Analysis and WGCNA

We conducted immunoinfiltration analysis using ImmuCellAI [12, 13]. The preprocessed gene expression matrices from GSE161533 and GSE23400 were imported into ImmuCellAI, and the “Analysis” function was utilized to quantify the infiltration of various immune cells in tumor samples.

Subsequently, based on the gene expression matrices and corresponding immunoinfiltration analysis results, we constructed a weighted gene co‐expression network using the R package “WGCNA (1.71)” and screened for gene modules associated with various immune cell infiltrations. Specifically, we first selected 10,000 genes with large standard deviations and identified outlier samples through sample clustering, followed by their removal. Then, we chose an appropriate soft threshold to construct the scale‐free network and TOM matrix. Next, we calculated the correlation between gene modules and immune cell infiltration using the “moduleTrait” function. If multiple associations were found between immune cells and gene modules, we applied two criteria for selection: (1) selecting immune cells with higher average infiltration abundance and (2) conducting correlation tests with *p* < 0.01. Subsequently, we obtained immune infiltration‐related genes by intersecting the gene modules obtained from GSE161533 and those obtained from GSE23400.

### 2.3 Enrichment Analysis and Core Gene Selection

We annotated the biological significance of intersecting genes using the R package “clusterProfiler (4.6.0)” to indirectly demonstrate the correlation between these genes and infiltrating immune cells. This annotation involved two enrichment analysis methods: Gene Ontology (GO) and Kyoto Encyclopedia of Genes (KEGG).

Subsequently, we imported the intersecting genes into the STRING database to construct a protein–protein interaction (PPI) network. Associations with the “combined score” less than 0.7 were excluded to obtain a reliable PPI network. The network was then imported into Cytoscape (3.9.2), and key genes in the network were calculated using the “MCC” method in the cytoHubba tool. Finally, the top ten ranked genes were selected as the core genes of the PPI network.

### 2.4 Gene Expression Differential Analysis and Survival Analysis

For chip data, we used the R package “limma (3.54.2)” to perform paired comparisons between ESCC samples and normal esophageal tissue samples from the GSE161533 and GSE23400 datasets to analyze gene expression differences. For high‐throughput sequencing data, we extracted RNA counts data for ESCC and normal esophageal tissue from the “UCSC Toil RNAseq Recompute Compendium” dataset and conducted differential analysis using the R package “DESeq2 (1.38.1)”.

As for survival analysis, we obtained data from TCGA. Utilizing the R package “survminer (0.4.9)”, we determined the optimal cutoff point and then assessed whether there were differences in overall survival (OS), disease‐specific survival (DSS), and progression‐free interval (PFI) between patients with high expression of core genes and those with low expression using the R package “survival (3.4.0)”.

Finally, we selected the core genes from the PPI network that were significant in both all expression differential analyses and all survival analyses as target genes. The immune cells corresponding to the gene module where the target genes were located were identified as target cells.

### 2.5 Mining of Single‐Cell RNA Data

First, we compared the expression differences of the target genes in different cells using the R package “Seurat(4.3.0)”. Subsequently, we explored whether the expression of the target genes was related to the differentiation and development of target cells using the R packages “IOBR(0.99.9)” and “Monocle(2.26.0)”. We also analyzed whether different tumor stages affect the expression of target genes in the target cells.

### 2.6 Validation of the Protein Expression of the Target Gene

We conducted western blot (WB) and immunohistochemistry (IHC) experiments to examine the protein expression of the target genes. The samples used in the experiments were obtained from ESCC patients, with detailed information provided in Table S3. All patients were unrelated Asians who were hospitalized and treated at the First Affiliated Hospital of Guangxi Medical University (Guangxi Province, China). ESCC was confirmed by histopathological examination of tissue obtained from surgical resection of the tumor or biopsy. None of the patients received chemotherapy or radiotherapy before tumor resection. Biological samples were collected immediately after tumor resection from patients and analyzed according to the specified procedures. This study was approved by the Ethics Committee of the First Affiliated Hospital of Guangxi Medical University, and informed consent was obtained from each patient. The experimental procedures are briefly described as follows.

For the WB, β‐actin (Affinity, AF7018, 1:9000) was used as the loading control, and ITGB2 (Abclonal, RA2173, 1:1000) as the target protein. SDS‐PAGE gels were first prepared, followed by protein extraction from tissue samples, sample denaturation, and electrophoresis. After electrophoresis, membrane transfer was performed using the wet transfer method. Finally, the membranes were scanned using a scanner, and the relative protein expression levels were assessed with ImageJ software.

For the IHC, ITGB2 (Abclonal, RA2173, 1:100) was used as the primary antibody. Formalin‐fixed, paraffin‐embedded tissue sections were first deparaffinized, followed by antigen retrieval. After removing endogenous peroxidase activity, the sections were blocked. Then, primary antibody incubation, secondary antibody incubation, and DAB staining were performed. Subsequently, counterstaining (nuclear staining), mounting, and imaging were carried out. Finally, ImageJ software was used to quantify the relative expression levels of ITGB2.

### 2.7 Validation of the Correlation Between Target Genes and Target Cells

For chip data, we extracted the infiltration abundance of target cells from the previous ImmuCellAI results, and then performed correlation analysis between the expression values of target genes extracted from the gene expression matrix and the infiltration abundance of target cells. For high‐throughput sequencing data, we first downloaded the immune infiltration data for esophageal cancer from the ImmuCellAI website, extracted the infiltration abundance of target cells in ESCC, and then converted the count data of ESCC to TPM data. Subsequently, we conducted correlation analysis between the TPM data of target genes and the infiltration abundance of target cells.

To enhance the reliability of the results, we additionally utilized six different algorithms from the TIMER2 website (TIMER, CIBERSORT, CIBERSORT.ABS, QUANTISEQ, XCELL, EPIC) to calculate the immune infiltration abundance of target cells in ESCC samples derived from TCGA. Subsequently, we performed correlation analysis between the results of these additional algorithms and the TPM data of target genes.

Subsequently, we utilized gene expression data from the GSE16533, GSE23400, and TCGA datasets to perform correlation analysis and assess the relationship between the target genes and the markers of target cells.

Finally, we performed dual immunofluorescence (IF) to simultaneously detect the expression of the target genes and the target cell markers in ESCC, providing further evidence for the association between the target gene and the target cells. Co‐localization analysis of the target genes expression and the target cell marker expression was conducted using the Coloc 2 plugin in ImageJ software. The experimental procedures are briefly described as follows.

The tissue section samples were obtained from ESCC patients, with detailed information provided in Table S3. The sources of the patients have been described previously. ITGB2 (Abcam, 10,554‐1‐AP, 1:50) and CD163 (Abcam, 68,218‐1‐lg, 1:50) were used as primary antibodies (target proteins). First, the tissue sections were deparaffinized, followed by antigen retrieval in either citrate buffer (pH 6.0) or Tris‐EDTA buffer (pH 9.0). Blocking was then performed using a blocking agent/antibody dilution solution. The sections were subsequently incubated with the primary antibodies. Immunostaining was visualized using tyramide signal amplification (TSA) with fluorophore‐conjugated secondary antibodies specific to each primary antibody. After each round of antibody staining, a microwave‐based stripping procedure was employed. Following staining, the sections were scanned and imaged using a microscope. Finally, ImageJ software was used to quantify the relative expression levels of ITGB2 and CD163. Co‐localization analysis of ITGB2 and CD163 expression was performed using the Coloc 2 plugin in ImageJ software.

### 2.8 Correlation Between Target Genes and Known Targets of Target Cells

We identified the primary known targets of the target cells through a review of relevant literature. Using gene expression data from GSE161533, GSE23400, and TPM data from TCGA, we conducted correlation analyses to determine the associations between these targets and the target genes.

### 2.9 Immunotherapy Response

We selected TME signature gene sets related to immunotherapy response based on relevant literature and gene sets collected by R package “IOBR (0.99.9)”. Utilizing the ESCC data from the GSE23400 dataset and the TPM data from TCGA, we quantified these TME signatures using the “ssGSEA” algorithm in the R package “IOBR(0.99.9)”. Subsequently, correlation analysis was conducted to understand the association between the target genes and these signatures.

### 2.10 Predicting Upstream miRNAs

Initially, we utilized miRWork to predict miRNAs that may potentially bind to the target genes. Subsequently, we filtered out miRNAs with a binding probability greater than 0.9. Differential expression analysis was conducted using data from GSE66274 and GSE67268 to identify miRNAs exhibiting differential expression between ESCC and esophageal normal tissues. Following this, we further refined the selection by filtering for miRNAs with an adjusted *p* < 0.01 and a fold change (log_2_FC) less than −2. Finally, we intersected the miRNAs obtained from GSE66274, those obtained from GSE67268, and those selected from miRWork to obtain the final miRNAs.

## 3 Results

### 3.1 Immune Infiltration Status in ESCC and WGCNA

**FIGURE 1:**
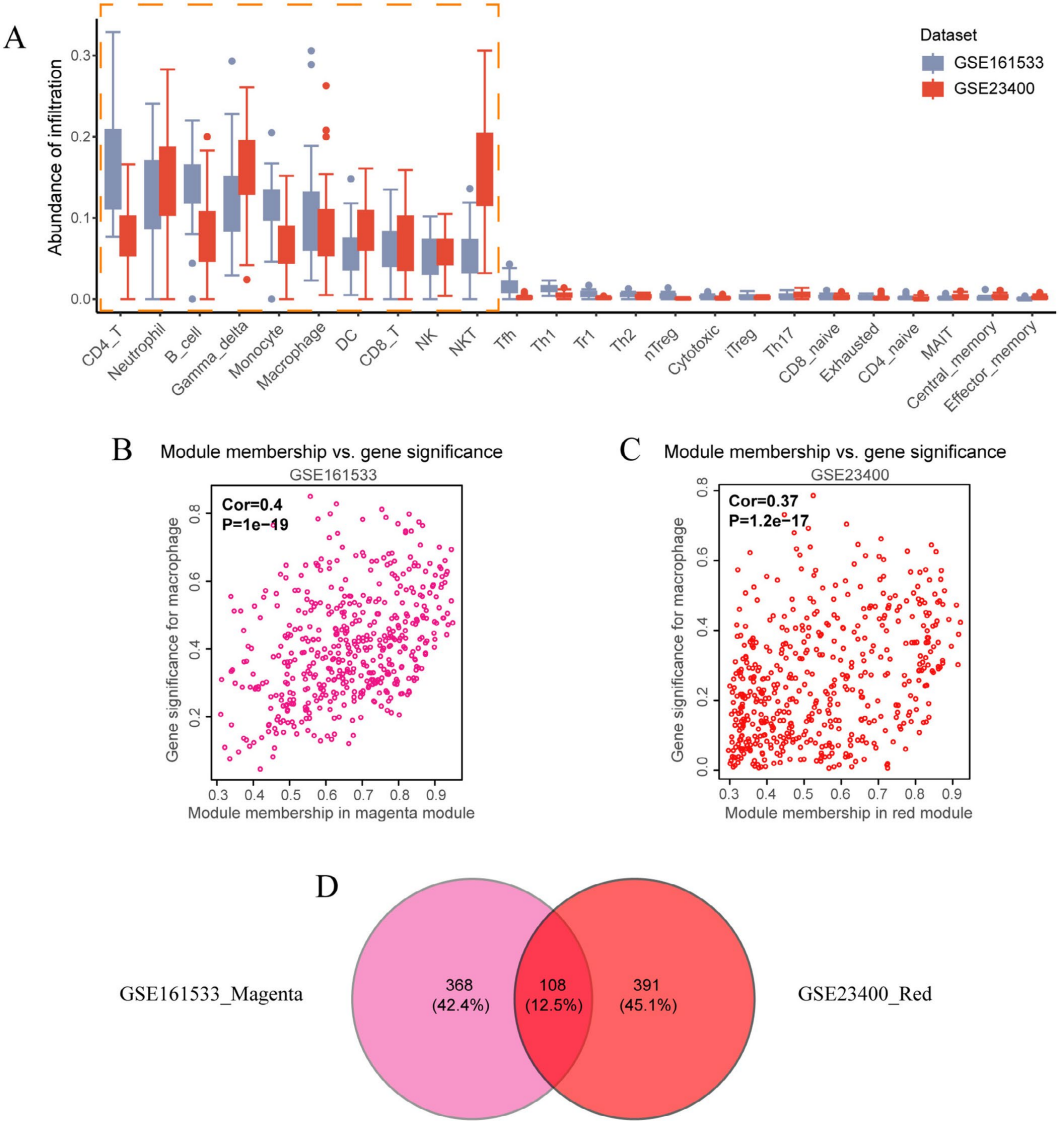
ImmuCellAI and WGCNA results. (A) Infiltration abundance of different immune cells in ESCC (Derived by ImmuneCellAI). (B) The correlation between the magenta module and macrophages in GSE161533. (C) The correlation between the red module and macrophages in GSE23400. (D) Venn diagram showing the intersection between the magenta module in GSE161533 and the red module in GSE23400.

The immune cell profiling using ImmuneCellAI revealed a significant presence of dendritic cells, B cells, macrophages, NK cells, and other immune cell types within ESCC (Figure 1A). However, the abundance of other immune cell types is comparatively lower. Consequently, these ten immune cell types were included for subsequent analysis.

For GSE161533, we set the “cutHeight” parameter of WGCNA to 95, removing 3 outlier samples (GSM4909616, GSM4909627, GSM4909622), and then selected a soft threshold of 6 to fit the optimal scale. At this point, *R* ^2^ = 0.901 (Figure S1A), and the average network connectivity was 33.5 (Figure S1C). After constructing the weighted gene co‐expression network and merging modules with correlation coefficients greater than 0.75, all genes were assigned to 16 different colored gene modules (Figure S1E). Through correlation analysis, we found multiple associations between gene modules and immune cell infiltration (Figure S2A), such as a significant correlation between the magenta module and macrophage infiltration (*p* = 0.001), and between the yellow module and NK cell infiltration (*p* = 0.001).

For GSE23400, we set the cutHeight to 65, removing 4 outlier samples (GSM573938, GSM573951, GSM573901, GSM573906), and then selected a soft threshold of 5 to fit the optimal scale. At this point, *R* ^2^ = 0.851 (Figure S1B), and the average network connectivity was 26.9 (Figure S1D). After constructing the weighted gene co‐expression network and merging modules with correlation coefficients greater than 0.75, all genes were assigned to 12 different colored gene modules (Figure S1F). Through correlation analysis, we also found multiple associations between gene modules and immune cell infiltration (Figure S2B), such as a significant correlation between the yellow module and neutrophil infiltration (*p* = 0.001), and between the red module and macrophage infiltration (*p* = 0.002).

Therefore, we screened using the two predefined filtering criteria and determined one gene module from each of the two sets of WGCNA results for further analysis. The magenta module was identified for GSE161533, and the red module for GSE23400 (Figure S2). The corresponding immune cell was macrophages. Subsequent analysis revealed a significant positive correlation between module membership and gene significance for both groups (Figure 1B,C). Thus, taking the intersection of the two gene modules yielded 108 genes associated with macrophage infiltration (Figure 1D).

### 3.2 Enrichment Analysis and PPI Network Screening

The outcomes of KEGG analysis suggested that the intersecting genes were linked to biological processes like “Phagosome,” “Osteoclast differentiation,” “Lysosome,” “NF‐kappa B signaling pathway,” and “Antigen processing and presentation” (Figure S3A). GO analysis results indicated that the intersecting genes were associated with biological characteristics including “macrophage activation,” “phagocytosis,” “cell activation involved in immune response,” “myeloid leukocyte activation,” “myeloid leukocyte migration,” “ficolin‐1‐rich granule,” “ficolin‐1‐rich granule lumen,” “phagocytic cup,” “endocytic vesicle membrane,” “integrin complex,” “mannose binding,” “immunoglobulin binding,” “immune receptor activity,” “pattern recognition receptor activity,” and “complement binding” (Figure S3B). These findings further emphasize the probable correlation between the expression of intersecting genes and macrophage infiltration in ESCC.

As shown in Figure S4A, using the STRING database, we obtained the protein–protein interaction (PPI) network of the relevant genes. Associations with a “combined score” less than 0.7 were excluded (Figure S4B), and using the “MCC” method of the cytoHubba tool for calculation, we ultimately identified ten core genes in the network (Figure S4C). Ranked from high to low based on their scores, they are: CSF1R, ITGB2, ITGAM, TYROBP, FCGR2A, CD14, FCER1G, ITGAX, TLR4, and C1QB.

### 3.3 Gene Expression Differential Analysis and Survival Analysis

**FIGURE 2:**
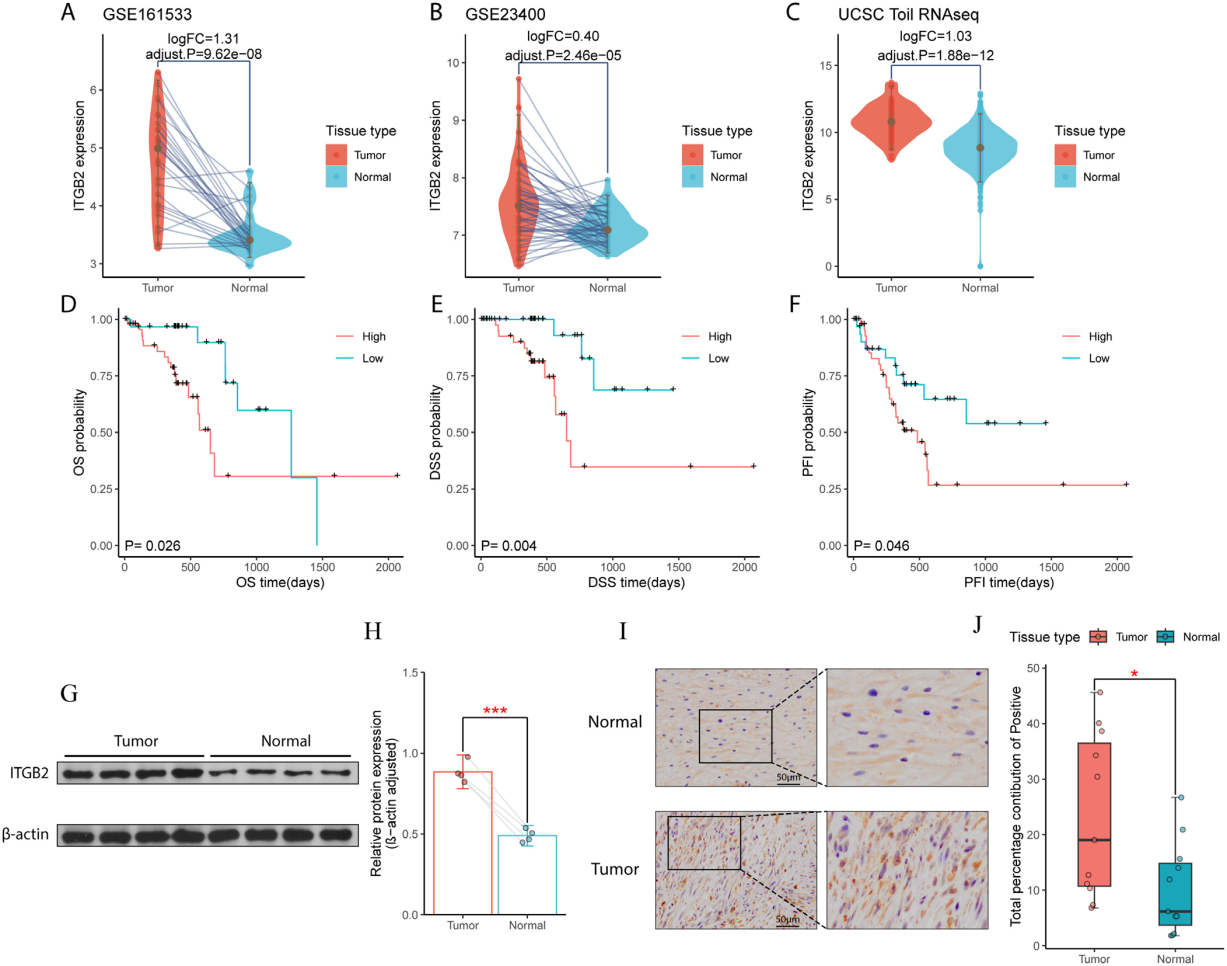
Expression differential analysis and survival analysis results for ITGB2. (A–C) Expression differential analysis results. (A) GSE161533. (B) GSE23400. (C) UCSC Toil RNAseq. (D–F) Kaplan–Meier curves for different survival metrics. (D) Overall survival (OS). (E) Disease‐specific survival (DSS). (F) Progression‐free interval (PFI). (G) WB results of tumor samples and normal tissue samples. (H) Differential analysis of WB based on quantification using ImageJ. (I) Representative IHC images of tumor samples and normal tissue samples. (J) Differential analysis of IHC based on quantification using ImageJ. * indicates *p* < 0.05, *** indicates *p* < 0.001.

Gene expression differential analysis and survival analysis revealed significance in ITGB2, ranked second among these ten core genes, in all analyses (Figure 2, Tables S4,S5). Results from three different datasets consistently showed higher expression of ITGB2 in ESCC compared to esophageal normal tissues (all *p* < 0.05, Figure 2A–C). Patients with high expression of ITGB2 in ESCC had shorter OS, DSS, PFI times compared to those with low expression of ITGB2 (all *p* < 0.05, Figure 2D–F).

Furthermore, both IHC and WB results demonstrated significant upregulation of ITGB2 protein in ESCC compared to esophageal normal tissues (all *p* < 0.05, Figure 2G–J).

These findings suggest that ITGB2 is a promising and worthy gene for further investigation. The overexpression of ITGB2 is likely associated with the occurrence and progression of ESCC, and this correlation may be closely related to macrophage infiltration. Therefore, we identified ITGB2 as the target gene for this study. Correspondingly, macrophages were identified as our target cells.

### 3.4 Parsing Single‐Cell RNA Data

**FIGURE 3:**
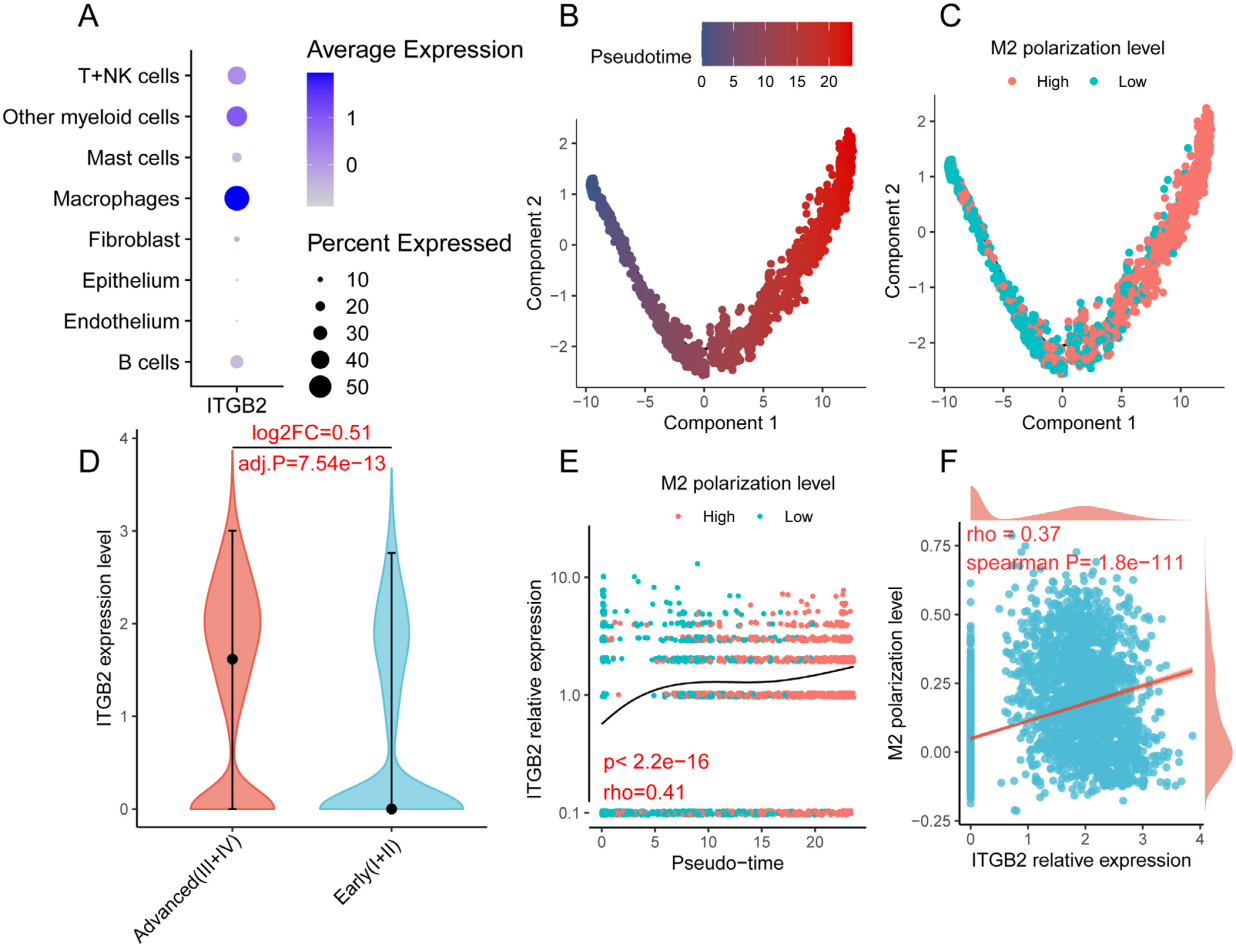
The analysis results of single‐cell sequencing data. (A) Expression profile of ITGB2 across different cell types. (B) Pseudotime analysis results of macrophages. (C) M2 polarization levels of macrophages at different time points. (D) Differential expression levels of ITGB2 in macrophages between early and late stage ESCC. (E) Correlation between macrophage development and intracellular expression levels of ITGB2. (F) Correlation between intracellular expression levels of ITGB2 and M2 polarization levels of macrophages.

The single‐cell sequencing samples comprised 20,793 cells. We classified cells using DCN, PDPN, CD2, CD3D, ITGAX, CD68, CD79A, KRT5 EPCAM, and TPSB2 as markers (Figure S5C). Subsequently, we isolated macrophages from myeloid cells using CD14 as a marker (Figure S5D), obtaining a total of 3491 macrophages (Figure S5A,B). We observed that ITGB2 expression in macrophages was higher than in other cell types (Figure 3A, Table S6).

We then performed pseudotime analysis on the macrophages. Following the widely accepted cancer immune editing theory [14], we assessed whether pseudotime conformed to an objective pattern; namely, macrophages in the TME would progressively acquire pro‐tumor characteristics. Over time, macrophages would exhibit increasingly significant M2 features. We quantified the strength of M2 features in each macrophage using the “ssGSEA” algorithm in the R package “IOBR.” Details of the M2 feature gene set are provided in Table S7.

The results confirm the reliability of our pseudotime analysis. As time progresses, the M2 characteristics of macrophages become more prominent (Figure 3B,C). We found a positive correlation between the expression of ITGB2 within macrophages and pseudotime, as well as M2 characteristics (all *p* < 0.05, Figure 3E,F). Further analysis revealed that in the microenvironment of advanced ESCC, ITGB2 expression within macrophages is higher compared to that in the microenvironment of early ESCC (adjusted *p* = 0.54e‐43, Figure 3D).

### 3.5 Examination of the Association Between ITGB2 and Macrophage Infiltration

**FIGURE 4:**
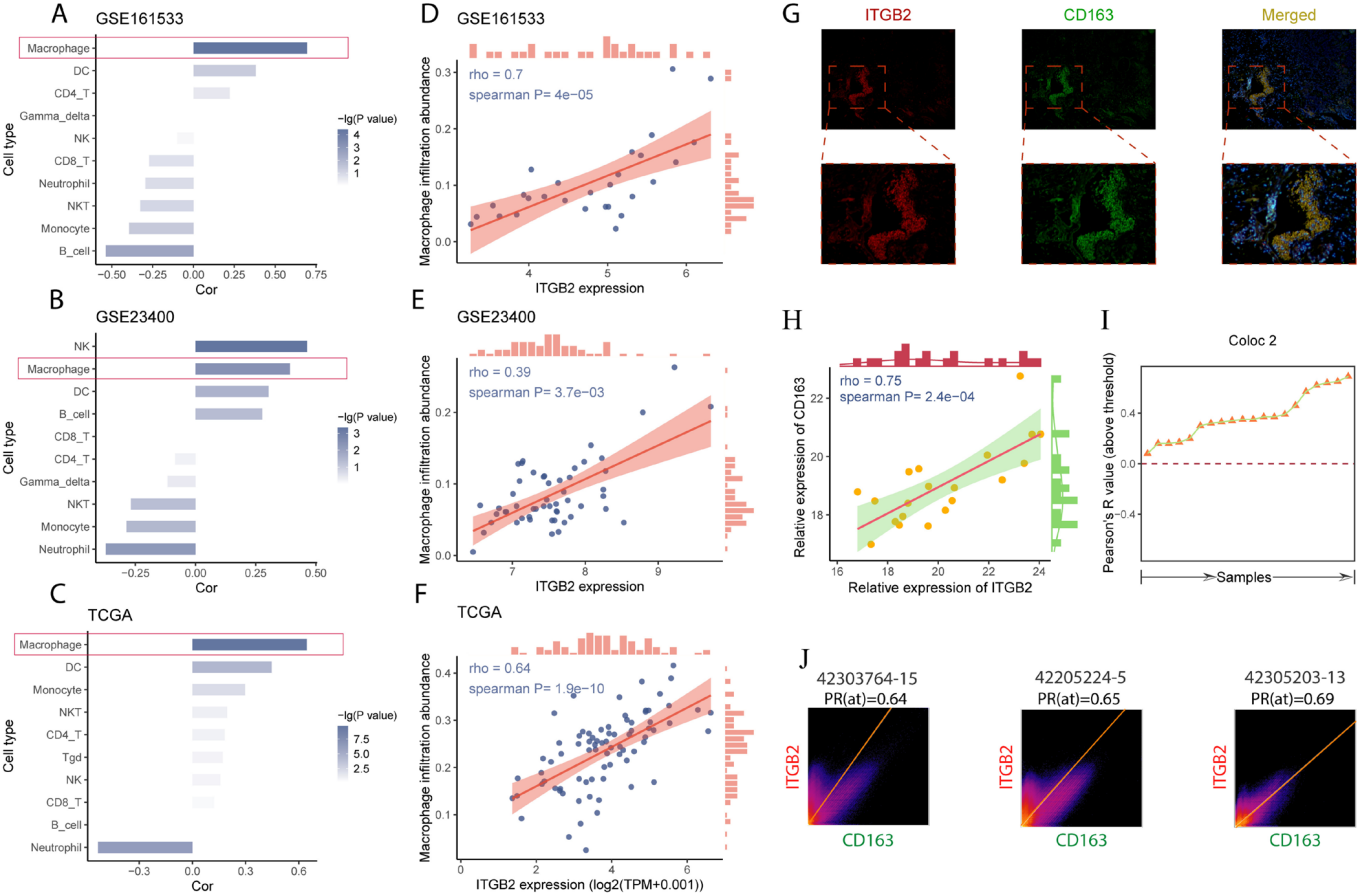
Analysis results of the correlation between the target gene ITGB2 and immune cell infiltration in ESCC, along with the results of dual immunofluorescence. (A–C) Correlation of ITGB2 expression with various immune cells across different datasets. (A) GSE161533. (B) GSE23400. (C) TCGA. (D–F) Correlation of ITGB2 expression with macrophage infiltration across different datasets. (D) GSE161533. (E) GSE23400. (F) TCGA. (G) Representative immunofluorescence images; red fluorescence represents ITGB2, and green fluorescence represents CD163; yellow light is produced by the overlap of red and green fluorescence. (H) Correlation analysis results between the fluorescence intensity of ITGB2 protein and the fluorescence intensity of CD163 protein. (I) Coloc2 results for all samples. (J) Representative co‐localization analysis results of all samples.

In all three different datasets, the expression of ITGB2 was significantly positively correlated with macrophage infiltration (*p* < 0.05, Figure 4A–C). In the GSE161533 dataset, the Spearman correlation coefficient (rho) was 0.7 with a *p*‐value of 4e‐05 (Figure 4D); in the GSE23400 dataset, the Spearman correlation coefficient was 0.39 with a *p*‐value of 3.7e‐03 (Figure 4E); in the TCGA dataset, the Spearman correlation coefficient was 0.64 with a *p*‐value of 1.9e−10 (Figure 4F). Results from multiple additional methods also indicated a significant positive correlation between ITGB2 expression and ESCC macrophage infiltration (Figure S6).

Subsequently, we analyzed the correlation between ITGB2 and M2 macrophage markers MRC1 (CD206) and CD163. We found that ITGB2 expression was significantly positively correlated with MRC1 and CD163 across different datasets (Table S8).

Finally, we performed dual IF to simultaneously detect the expression of ITGB2 and CD163 proteins in ESCC tissues (Figure 4G). Correlation analysis revealed that the results were consistent with those from the three datasets mentioned above, showing a significant positive correlation between ITGB2 and CD163 expression (Figure 4H). Using the Coloc 2 plugin in ImageJ software, we further identified a co‐localization relationship between ITGB2 and CD163 expression (Figure 4I,J). These findings further support the association between ITGB2 and macrophage infiltration in ESCC.

### 3.6 Correlation of ITGB2 With Reported Targets of Macrophages

**FIGURE 5:**
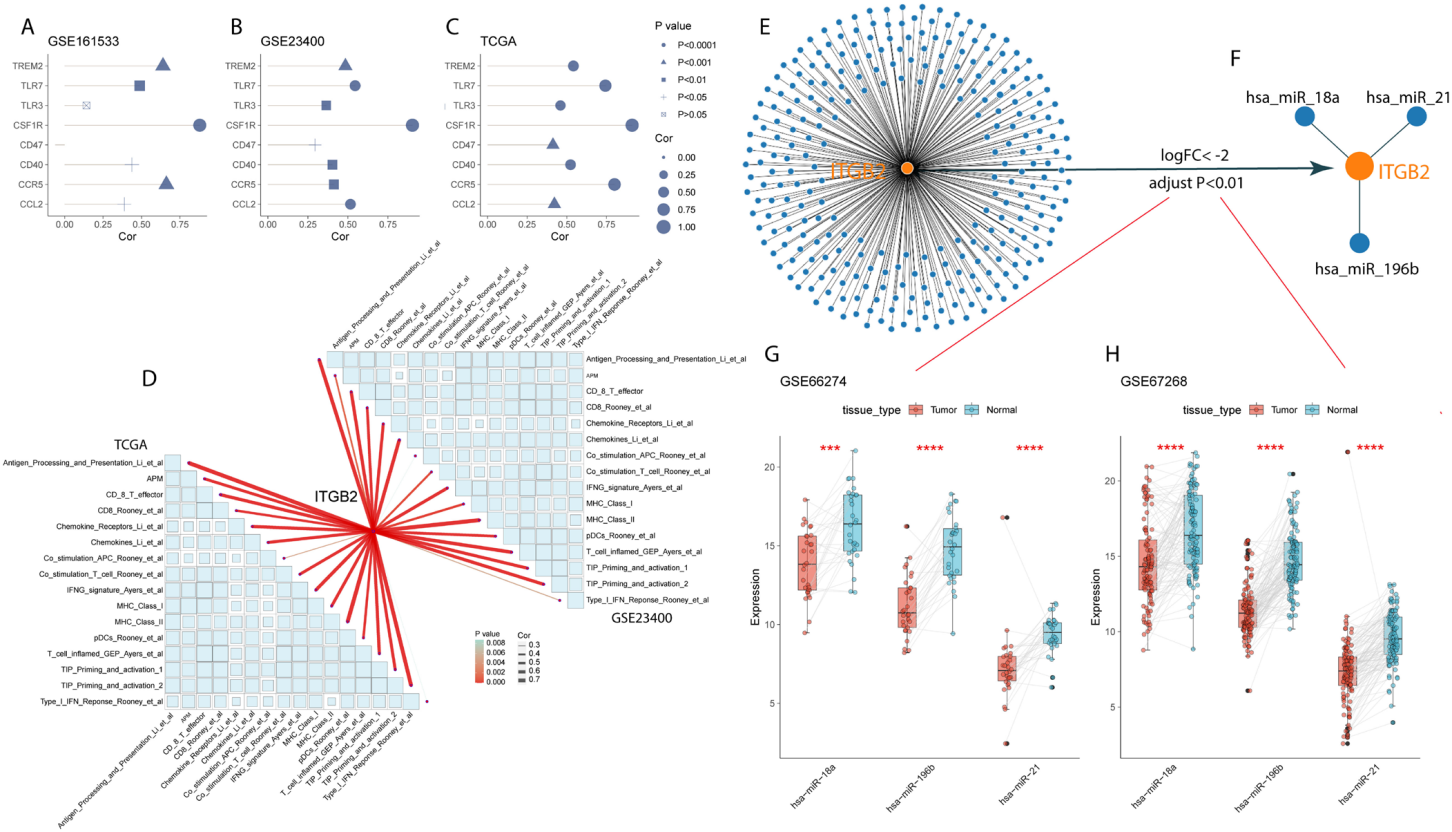
Analysis results of the correlation between the target gene ITGB2 and macrophage markers, its association with immunotherapy, and the exploration of upstream miRNAs. (A–C) The correlation between ITGB2 and known primary targets of macrophages in different datasets. (A) GSE161533. (B) GSE23400. (C) TCGA. (D) The correlation between ITGB2 expression in ESCC and 16 immune therapeutic response‐related TME signatures. (E) miRNAs with a binding probability greater than 0.9 in miRWork. (F) Key miRNAs obtained through screening. (G) Differential expression of key miRNAs between ESCC and normal esophageal tissues in GSE66274. (H) Differential expression of key miRNAs between ESCC and normal esophageal tissues in GSE67268.

Through a review of relevant literature, we identified ten major known targets of macrophages, namely CCL2, CCR5, CSF1R, CD47, CD40, TLR3, TLR7, and TREM2. Clinical trials related to these targets are currently underway, with some having achieved certain results (Table S11). In the GSE161533 dataset, we found that ITGB2 expression was positively correlated with these targets (all correlation coefficients greater than 0, all *p* < 0.05), except for TLR3 (*p* > 0.05) (Figure 5A). In the GSE23400 and TCGA datasets, ITGB2 expression was positively correlated with all targets (all correlation coefficients greater than 0, and all *p* < 0.05) (Figure 5B,C).

### 3.7 Immunotherapy Response

By reviewing relevant literature and referring to various gene sets collected by “IOBR”, we screened 16 gene sets associated with tumor immunotherapy response (Table S9). Through correlation analysis, we found that the expression of ITGB2 is positively correlated with these 16 TME features related to immunotherapy response, in both the GSE23400 and TCGA datasets (all *p* < 0.05, correlation coefficients > 0, Figure 5D, Table S10).

### 3.8 Upstream miRNA

Utilizing miRWork, we identified 2796 miRNAs with a binding probability greater than 0.9 to ITGB2 (Figure 5E). Through the aforementioned screening methods, we ultimately identified three key miRNAs (hsa‐miR‐18a, hsa‐miR‐196a, hsa‐miR‐21, Figure 5F). In the GSE66274 dataset, compared to esophageal normal tissues, these three key miRNAs were downregulated in ESCC (all *p* < 0.001, Figure 5G). Similar results were observed in the GSE67268 dataset (all *p* < 0.0001, Figure 5H).

## 4 Discussion

First and foremost, it is undeniable that there are some differences between the quantification of ESCC immune infiltration by ImmuCellAI and the cell clustering results from scRNA, owing to technical disparities. However, it is equally undeniable that both techniques' outcomes indicate a rich infiltration of macrophages in ESCC. Presently, due to the heterogeneity and complexity of the TME [15], the application and efficacy of immunotherapy in ESCC remain limited. Nevertheless, research has shown that tumor tissue‐infiltrating macrophages can influence tumor development through multiple mechanisms, underscoring their significant research and potential clinical utility [15, 16].

ITGB2 (integrin subunit beta 2) is the beta subunit of β2 integrins. ITGB2 can form four different types of β2 integrins with four different alpha subunits (CD11a, CD11b, CD11c, CD11d). The functions of the four types of β2 integrins are mostly similar [17]. Initially discovered to be expressed in leukocytes, ITGB2 promotes leukocyte adhesion to endothelial cells, leading to extravasation [18]. Nowadays, with the continuous deepening of research, ITGB2 has been found to be associated with some cancers. For example, Paierhati et al. found that the expression level of ITGB2 in TNBC (Triple‐Negative Breast Cancer) is significantly higher than in normal breast tissue, and high expression of ITGB2 in TNBC affects patient prognosis [19]. Xu et al. suggested that ITGB2 could serve as a novel prognostic factor for clinical outcomes and immune therapy response in gliomas, and it could also be a target for immune therapy in glioma patients [20].

In this study, we employed various bioinformatics approaches to identify ITGB2 as a gene closely associated with macrophage infiltration in ESCC. Previously, Yao et al. reported a positive correlation between ITGB2 and macrophage infiltration in ESCC [21]. However, their study only used the CIBERSORT algorithm to assess macrophage infiltration in a single dataset, and they did not clarify whether the ESCC samples were from TCGA or the GSE23400 dataset. In our research, we quantified macrophage infiltration in the GSE161533, GSE23400, and TCGA datasets using ImmuCellAI. Additionally, we utilized six other algorithms (TIMER, CIBERSORT, CIBERSORT.ABS, QUANTISEQ, XCELL, EPIC) to quantify macrophage infiltration in TCGA's ESCC samples. The most of the results indicated a positive correlation between ITGB2 expression and macrophage infiltration in ESCC. Subsequently, we confirmed the positive correlation between ITGB2 expression and M2 macrophage markers MRC1 (CD206) and CD163 using the GSE161533, GSE23400, and TCGA datasets. Additionally, dual immunofluorescence provided further evidence of the positive correlation between ITGB2 protein expression and the M2 macrophage marker CD163 protein expression in ESCC, with co‐localization observed between ITGB2 and CD163. These findings further substantiate the positive correlation between macrophage infiltration, particularly M2 macrophages, and ITGB2 expression in ESCC, significantly enhancing the reliability of our conclusions.

Moreover, during the screening process and further analysis, we obtained additional meaningful results related to it.

Firstly, the results from three distinct datasets consistently demonstrate the overexpression of ITGB2 in ESCC. Additionally, IHC and WB results indicate higher protein levels of ITGB2 in ESCC compared to normal esophageal tissues. Furthermore, ESCC patients with high ITGB2 expression exhibit shorter overall survival (OS), disease‐specific survival (DSS), and progression‐free interval (PFI) compared to those with low ITGB2 expression. These findings strongly support the reliability of ITGB2 overexpression in ESCC and its significant potential as a prognostic biomarker.

Secondly, through the exploration of single‐cell sequencing data, we discovered that within the microenvironment of ESCC, the expression of ITGB2 in macrophages is significantly higher compared to other cells. It is well‐known that under the influence of the tumor microenvironment, macrophages continually evolve towards a tumor‐promoting phenotype [14]. Through pseudotime analysis, we observed a positive correlation between ITGB2 expression and the development of macrophages, as well as M2 characteristics. This indicates that the expression of ITGB2 within macrophages increases progressively as they evolve towards a tumor‐promoting phenotype. This undoubtedly represents a novel finding, offering new insights for targeted immunotherapy directed at macrophages. Additionally, we observed that in the microenvironment of advanced‐stage ESCC, the expression of ITGB2 in macrophages is higher compared to early‐stage ESCC. This finding also suggests that with ESCC progression, the expression of ITGB2 within macrophages increases.

Third, we found that most correlation analyses indicated a positive correlation between ITGB2 expression and macrophage targets currently in clinical trials. This suggests that higher ITGB2 expression may enhance the feasibility of targeting macrophages. We further explored the potential of using ITGB2 to evaluate the response to immunotherapy. Our findings revealed that ITGB2 is positively correlated with 16 TME features associated with immunotherapy response, suggesting that ITGB2 may be a viable marker for assessing the immunotherapy response in ESCC patients. Higher ITGB2 expression in patients could indicate a greater likelihood of benefiting from immunotherapy. Additionally, we identified three miRNAs associated with abnormal ITGB2 expression, providing a reference for further exploration of upstream molecules influencing ITGB2 expression.

Macrophages infiltrating tumor tissues are also known as TAMs (tumor‐associated macrophages). It is well established that macrophages exhibit a high degree of plasticity, allowing them to adopt a wide range of phenotypes in response to different cytokine environments and surrounding tissue conditions [22, 23]. Despite the complexity and diversity of macrophage activation states, they are generally categorized into two main types: M1 classically activated macrophages and M2 alternatively activated macrophages [24]. M1 macrophages are associated with anti‐tumor functions, capable of phagocytosing cancer cells and recruiting T cells (pro‐inflammatory) [25, 26]. In contrast, M2 macrophages are linked to immunosuppressive functions (anti‐inflammatory), promoting tumor growth and metastasis through various mechanisms [27]. According to the widely accepted concept of cancer immunoediting [28], during the phases of immune surveillance and elimination, the immune system can control cancer development by successfully recognizing and eradicating cancer cells. At this stage, macrophages play a crucial role due to their ability to mediate the phagocytosis and clearance of cancer cells and present cancer neoantigens to T cells (M1 phenotype) [29]. However, under immune‐mediated pressure, cancer cells undergo immunoediting to evade immune recognition. To support tumor progression, they continuously recruit circulating monocytes and tissue‐resident macrophages into the TME and polarize them into tumor‐promoting (M2) macrophages by secreting various soluble and mechanical factors [14, 30].

Therefore, the core of macrophage‐based tumor therapy lies in reducing anti‐inflammatory (tumor‐promoting) macrophages and/or increasing pro‐inflammatory (anti‐tumor) macrophages [14, 30]. However, a critical challenge in this approach remains: how to achieve tumor‐specific outcomes without compromising the responses of healthy innate and adaptive immune cells [14].

For example, blocking the CSF1/CSF1R axis is currently a well‐established method to eliminate existing TAMs or inhibit their recruitment [31, 32]. However, the effectiveness of this approach has been limited. There are two main reasons for this: (1) Blocking CSF1/CSF1R can lead to compensatory mechanisms, such as increased signaling through alternative survival pathways or enhanced activity of Tregs in the TME [33, 34]; (2) CSF1/CSF1R blockade may result in the depletion of tissue‐resident macrophages, which are essential for maintaining tissue homeostasis, as their survival depends on CSF1R signaling [35].

Therefore, as we mentioned in the introduction, there is a need for further exploration of tumor immunology‐related molecular mechanisms and therapeutic strategies. Our findings offer a potential new approach for the immunotherapy of ESCC.

Previous studies have suggested that β2 integrin may be involved in macrophage differentiation [17]. It is known that osteoclast progenitor cells express MAC‐1 (CD11b/ITGB2), and macrophages can transdifferentiate into osteoclasts [17]. Research by Kyung‐Hyun et al. showed that, compared to wild‐type (WT) mice, MAC‐1‐deficient mice exhibited greater bone loss, accompanied by an increased number of osteoclasts [36]. In our study, we found that ITGB2 expression in ESCC‐infiltrating macrophages increases as these macrophages develop towards a tumor‐promoting (M2) phenotype. After ESCC progression, ITGB2 expression in these macrophages becomes elevated. This suggests that inhibiting ITGB2 expression might prevent the macrophages from adopting a tumor‐promoting role, although further experimental validation is required to confirm this hypothesis.

However, we believe that merely inhibiting ITGB2 expression in the body may not be an appropriate measure. This approach does not address the challenges previously mentioned and could potentially lead to a range of complications [17]. Inducing M1 macrophages in vitro and then knocking out ITGB2 may be a more suitable strategy. If conditions permit, this will be a key focus of our future research.

Actually, in an early study, the Andreesen team in Germany administered monocyte‐derived macrophage therapy to 15 patients with advanced‐stage cancer who had not responded to standard treatments. Monocytes were collected via leukapheresis and cultured with autologous serum for 7 days to induce their differentiation into macrophages. Before injection into patients, these macrophages were “educated” with IFNγ to induce an M1 phenotype. The macrophages were then administered to patients via intravenous or intraperitoneal injection, with each dose containing up to 1.7 × 10^9^ cells. Although no significant reduction in the size of primary tumors was observed, some patients experienced stable disease for up to 6 months following treatment. Among the seven peritoneal carcinoma patients who received intraperitoneal macrophage injections, two showed a complete resolution of ascites. Seven out of the 15 patients exhibited elevated serum IL‐6 levels, indicating an induced inflammatory response. Importantly, no adverse effects were reported apart from mild fever and abdominal discomfort associated with intraperitoneal injections [37]. Subsequent studies using a similar protocol to generate IFNγ‐activated macrophages, known as macrophage‐activated killer (MAK) cells, demonstrated the antitumor activity of these cells in vitro and in preclinical models. Notably, Ritchie and colleagues used 111In‐oxine radiolabeling to track MAK cells and demonstrated that these “educated” macrophages actively migrated to metastatic sites in patients with metastatic ovarian cancer [38]. This migration was induced by both intravenous and intraperitoneal injections, with a higher proportion of patients showing migration following intraperitoneal administration. The injection of macrophages appeared to be safe, with no reports of treatment‐related high‐grade toxicities.

Although significant clinical efficacy was lacking, these studies did provide critical insights into the development of macrophage therapy. Firstly, dose‐escalation studies did not reveal significant toxicity associated with M1 macrophage injections. The most common side effects were mild fever and discomfort at the injection site. However, due to the absence of clinical response, higher therapeutic levels of MAK than those used in these studies may be required. While the limited efficacy observed in these trials was not extensively investigated, it is plausible that the endogenous antitumor activity of IFNγ‐activated macrophages was insufficient to drive meaningful responses. Notably, since macrophage polarization is a dynamic process influenced by external signals, the TME may have induced a shift of the infused IFNγ‐primed M1 macrophages towards an M2 phenotype. This suggests that more sustained methods of inducing M1 macrophage polarization may be necessary in the future [30].

Based on our findings, knocking out ITGB2 may potentially prevent or slow the polarization of macrophages towards the M2 phenotype. Therefore, a feasible novel approach could involve inducing M1 macrophages in vitro, followed by ITGB2 knockout, and subsequently reinfusing the ITGB2‐deficient M1 macrophages into patients. However, this remains a hypothesis that still requires future experimental evidence for validation.

In summary, this study thoroughly confirmed the positive correlation between macrophage infiltration and ITGB2 expression in ESCC through the integration of various data, methods, and experiments. ITGB2 is overexpressed in ESCC and holds potential as a prognostic biomarker for the disease. For the first time, we proposed that ITGB2 expression in infiltrating macrophages within ESCC increases as these macrophages undergo tumor‐promoting polarization. Following ESCC progression, the expression of ITGB2 in infiltrating macrophages is elevated. The higher the ITGB2 expression, the greater the feasibility of targeting macrophages; additionally, we found that assessing ESCC patients' immune responses to therapy via ITGB2 expression is viable. Moreover, we identified three miRNAs associated with aberrant ITGB2 expression, providing a reference for further exploration of upstream molecular interactions with ITGB2. Finally, based on our findings and previous studies, we propose a meaningful hypothesis: inducing M1 macrophages in vitro, followed by ITGB2 knockout, and then reinfusing these ITGB2‐deficient M1 macrophages into patients may represent a feasible new immunotherapeutic approach, offering a novel strategy for ESCC immunotherapy.

## Author Contributions

**Tao Huang:** conceptualization (equal), data curation (equal), formal analysis (equal), methodology (equal), resources (equal), software (equal), validation (equal), visualization (equal), writing – original draft (equal), writing – review and editing (equal). **Longqian Wei:** data curation (equal), investigation (equal), resources (equal), validation (equal), writing – original draft (supporting). **Huafu Zhou:** conceptualization (lead), funding acquisition (lead), investigation (lead), project administration (lead), resources (lead), supervision (lead), writing – review and editing (lead). **Jun Liu:** data curation (equal), funding acquisition (equal), investigation (equal), project administration (equal), resources (equal), validation (equal), visualization (equal), writing – review and editing (equal).

## Ethics Statement

This study was approved by the Ethics Committee of the First Affiliated Hospital of Guangxi Medical University, and informed consent was obtained from each patient.

## Conflicts of Interest

The authors declare no conflicts of interest.

## Supporting information


Figure S1.

Table S1.

## Data Availability

The publicly available data can be accessed through the respective website. Processed data and code can be obtained from the corresponding author upon request.
