# Supplementary material for: Macrophage Infiltration and ITGB2 Expression in ESCC: A Novel Correlation
Source: Cancer Med. 2025 Jan 17;14(2):e70604. doi: 10.1002/cam4.70604 (PMC11742006; doi:10.1002/cam4.70604)
Supplement: Supplementary file 1 — Figure S1. [file CAM4-14-e70604-s002.docx]

Supplementary Figures for

macrophage infiltration and ITGB2 Expression in ESCC: a novel correlation

Tao Huang,Longqian Wei, Huafu Zhou, Jun Liu

Correspondence to: lj_gxmuyfy_c_t_s@163.com

**This file contains the following content:**

| **Contents** | Description |
| --- | --- |
| **Supplementary Fig. 1** | Weighted gene co-expression network analysis. |
| **Supplementary Fig. 2** | Correlation between gene modules and immune cells. |
| **Supplementary Fig. 3** | Enrichment analysis results of genes related to macrophage infiltration. |
| **Supplementary Fig. 4** | Selection process for core genes. |
| **Supplementary Fig. 5** | Single-cell RNA sequencing data cell clustering results. |
| **Supplementary Fig. 6** | Correlations between ITGB2 and ESCC macrophage infiltration calculated by additional various algorithms. |


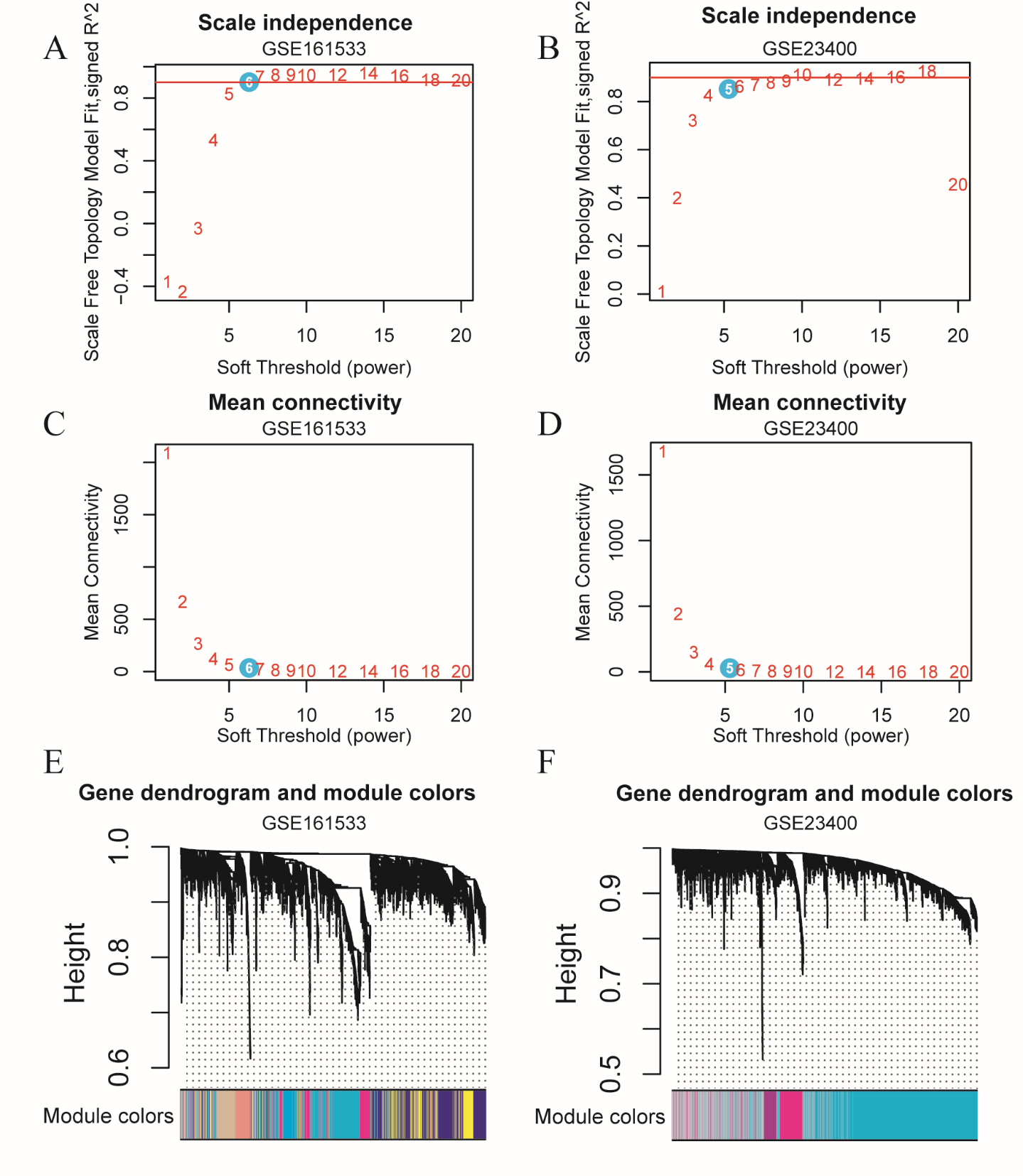


Supplementary Fig.1 Weighted gene co-expression network analysis.

(A-B) Scale independence plots. (A) for GSE161533, (B) for GSE23400. (C-D) Mean connectivity plots. (C) for GSE161533, (D) for GSE23400. (E-F) Gene clustering dendrograms. (E) for GSE161533, (F) for GSE23400.


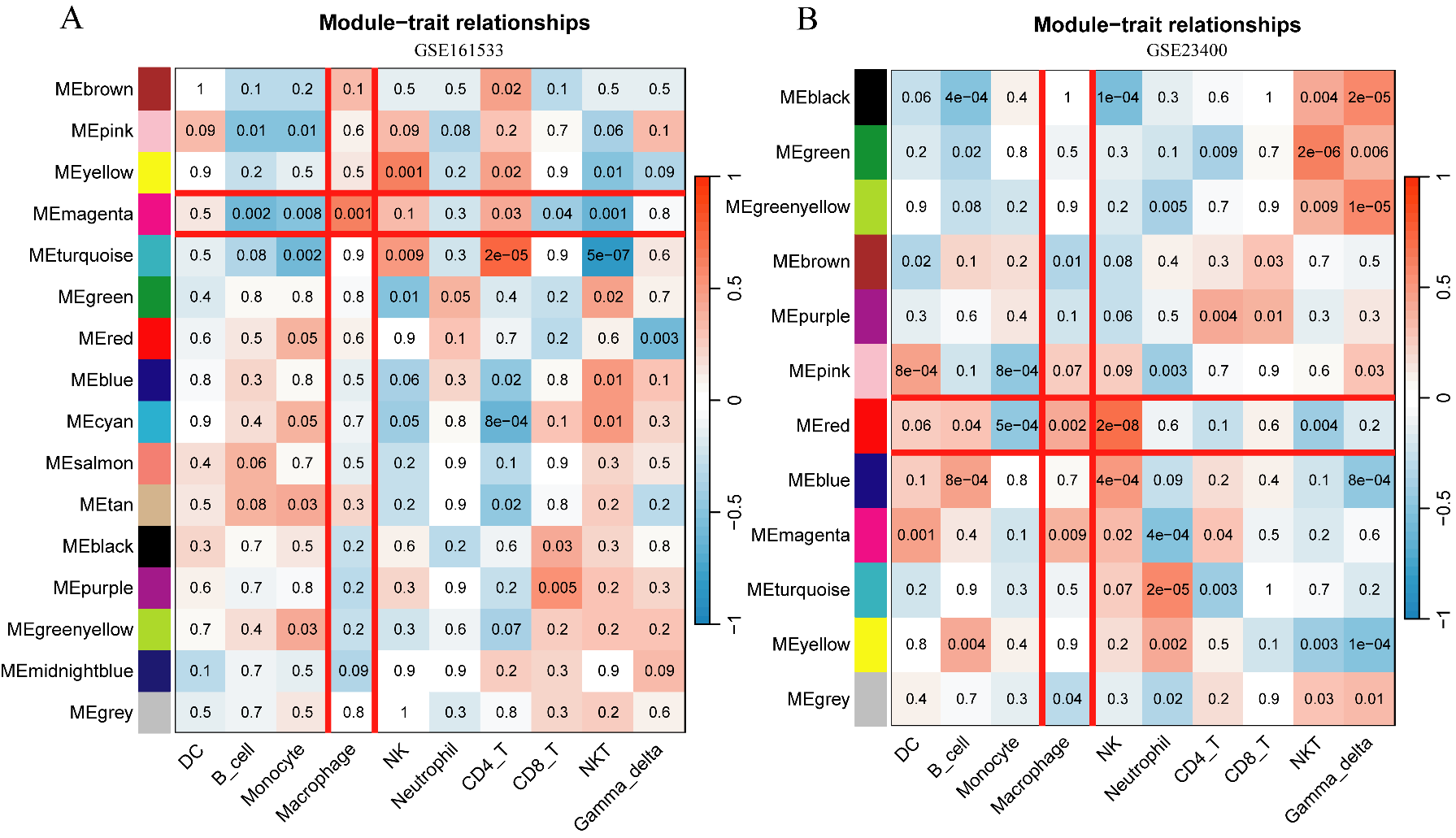


Supplementary Fig. 2 Correlation between gene modules and immune cells.

(A)represents the GSE161533 dataset ,(B) represents the GSE23400 dataset.


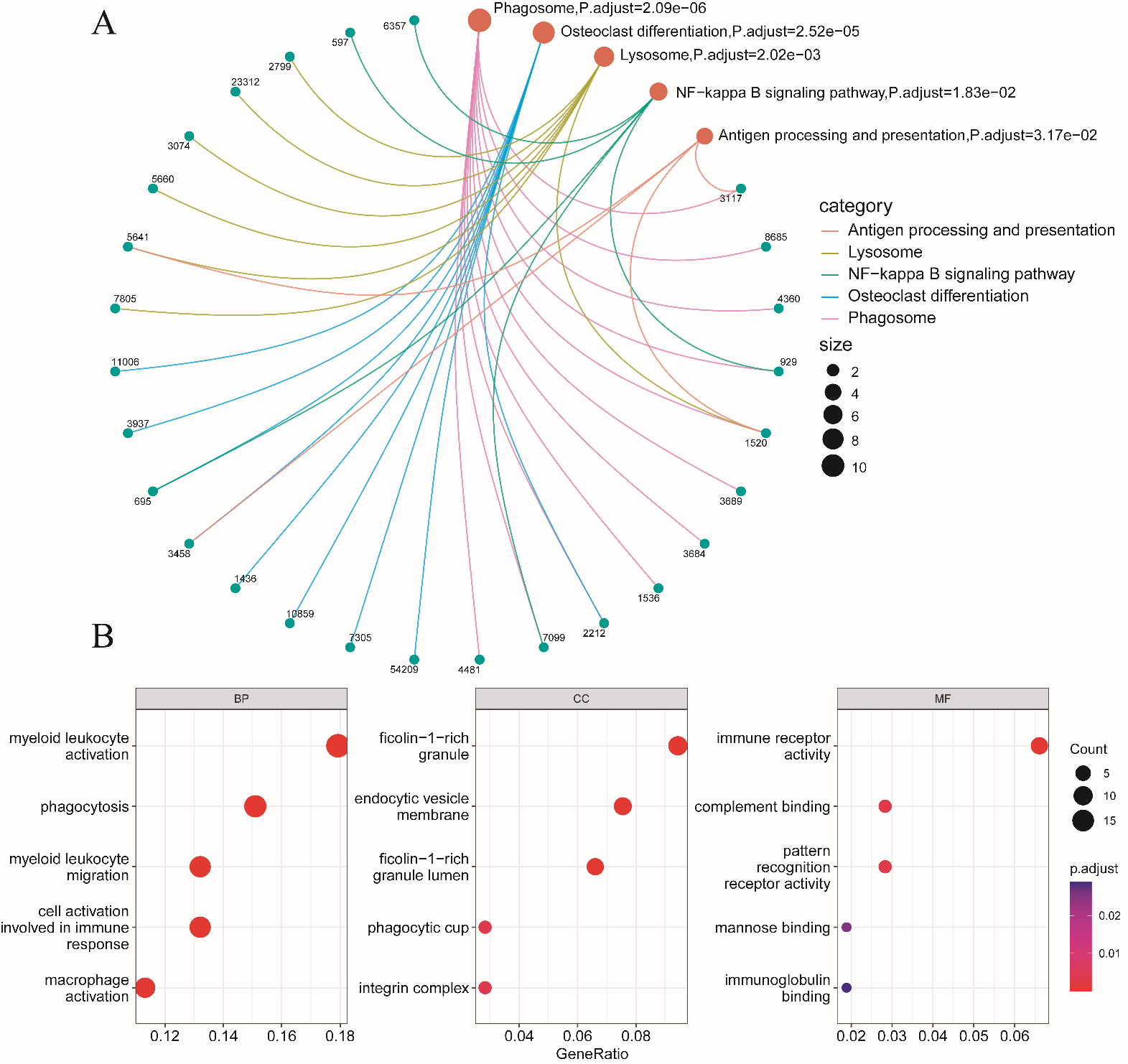


Supplementary Fig. 3 Enrichment analysis results of genes related to macrophage infiltration.

1. shows the KEGG enrichment analysis, and (B) shows the GO enrichment analysis.


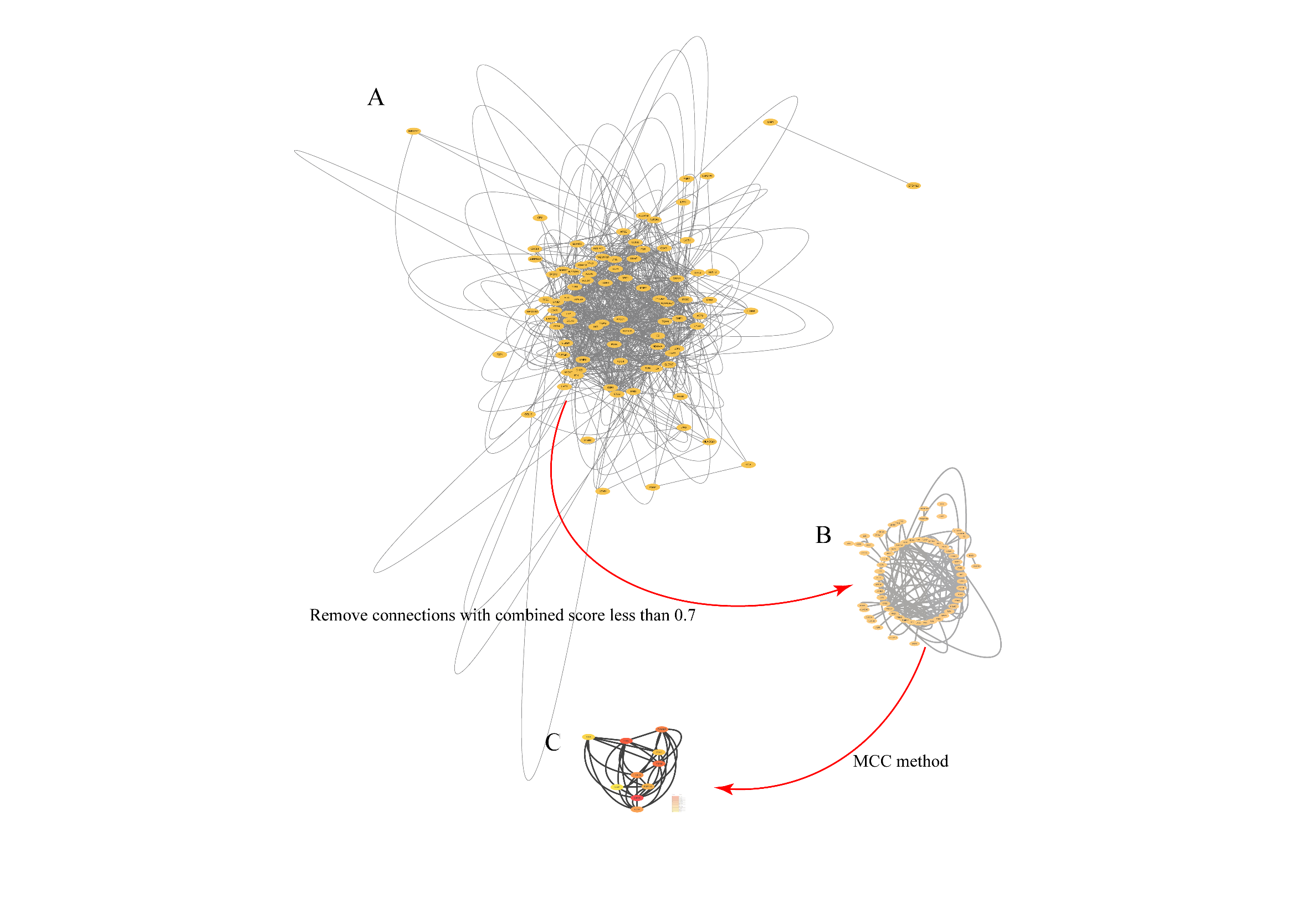


Supplementary Fig. 4 Selection process for core genes.

(A) shows the PPI network obtained through STRING database. (B) shows the reliable network after removing connections with a combined score less than 0.7. (C) displays the core network obtained through the MMC method.


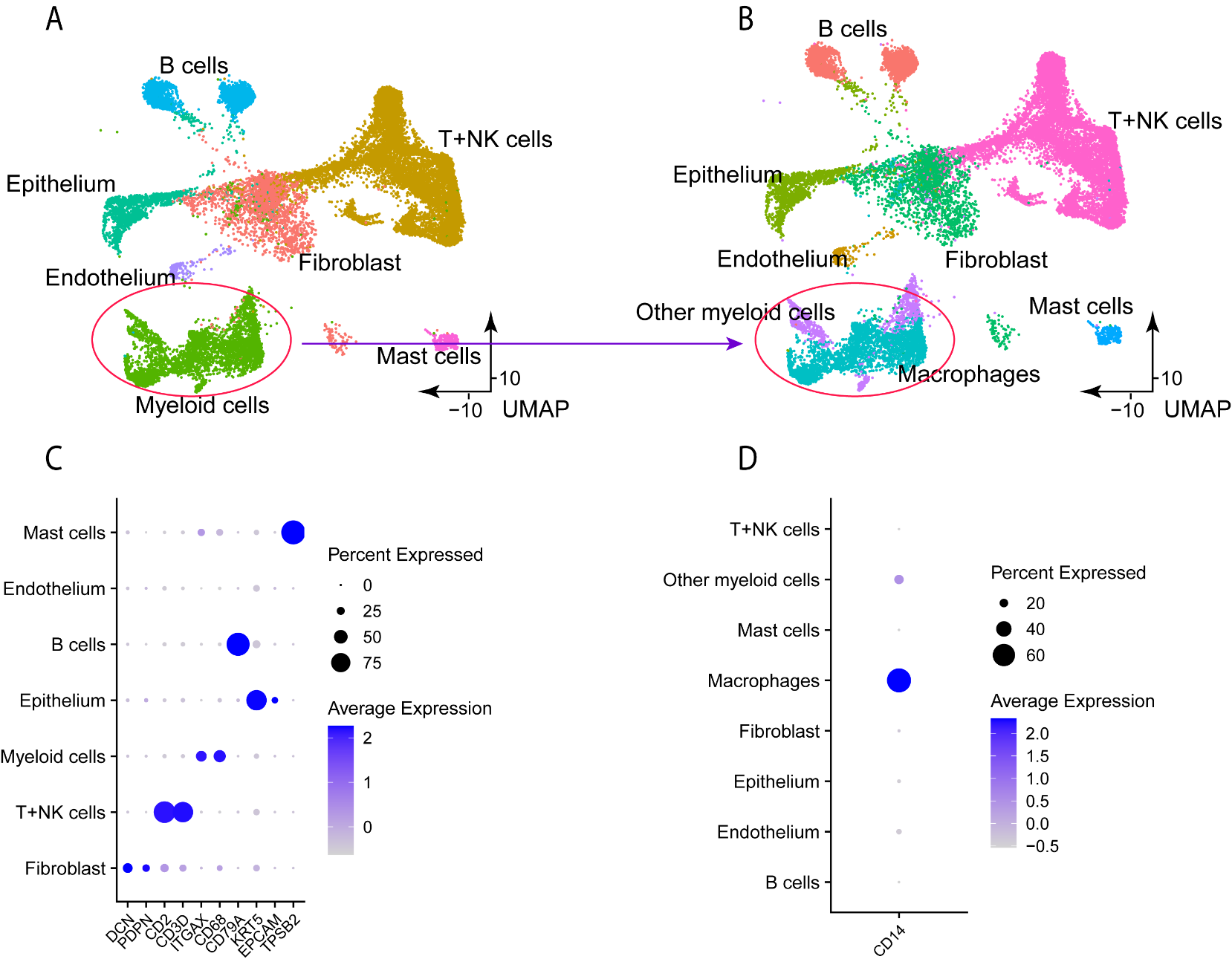


Supplementary Fig. 5 Single-cell RNA sequencing data cell clustering results.

（A） Cell clustering results before separating macrophages from myeloid cells.(B)Cell clustering results after separating macrophages from myeloid cells.(C)Expression patterns of markers used for cell clustering across different cell types.(D) The expression pattern of the macrophage marker CD14 across various cell types.


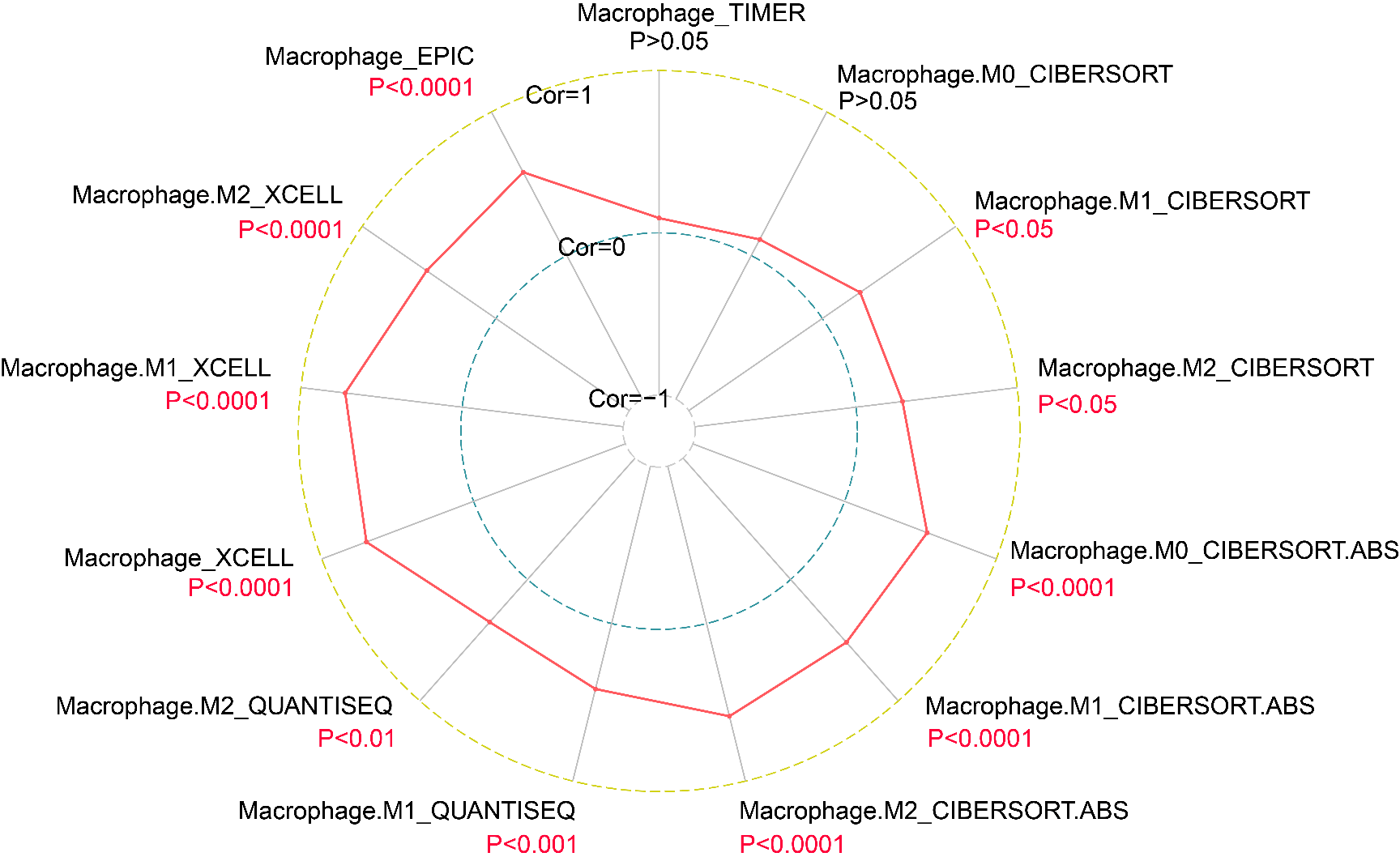


Supplementary Fig. 6 Correlations between ITGB2 and ESCC macrophage infiltration calculated by additional various algorithms.
