# Supplementary material for: Macrophage Infiltration and ITGB2 Expression in ESCC: A Novel Correlation
Source: Cancer Med. 2025 Jan 17;14(2):e70604. doi: 10.1002/cam4.70604 (PMC11742006; doi:10.1002/cam4.70604)
Supplement: Supplementary file 2 — Table S1. [file CAM4-14-e70604-s001.docx]

Supplementary Tables for

macrophage infiltration and ITGB2 Expression in ESCC: a novel correlation

Tao Huang,Longqian Wei, Huafu Zhou, Jun Liu

Correspondence to: lj_gxmuyfy_c_t_s@163.com

**This file contains the following content:**

| **Contents** | Description |
| --- | --- |
| **Supplementary Table 1** | The types and sources of data for this study. |
| **Supplementary Table 2** | Characteristics of patients with ESCC in GSE199619. |
| **Supplementary Table 3** | Summary of samples’ information used in the experiments. |
| **Supplementary Table 4** | Differential gene expression analysis results of core genes in the PPI network. |
| **Supplementary Table 5** | Survival analysis results of core genes in the PPI network. |
| **Supplementary Table 6** | The expression of ITGB2 in macrophages compared to other cells. |
| **Supplementary Table 7** | M2 polarization signature gene set. |
| **Supplementary Table 8** | Correlation between ITGB2 and M2 Macrophage markers. |
| **Supplementary Table 9** | The origin of the 16 TME gene sets related to immunotherapy response. |
| **Supplementary Table 10** | The correlation of ITGB2 with 16 TME features related to immunotherapy response. |
| **Supplementary Table 11** | Clinical trials relevant to the selected macrophage targets in this study. |

| Table S1. The types and sources of data for this study | | | |
| --- | --- | --- | --- |
| Data type | Source (dataset) | Sample type | Number of samples |
| Gene chip | GSE161533 | Normal tissue | 28 |
| Gene chip | GSE161533 | Tumor | 28 |
| Gene chip | GSE23400 | Normal tissue | 53 |
| Gene chip | GSE23400 | Tumor | 53 |
| Gene chip | GSE66274 | Normal tissue | 30 |
| Gene chip | GSE66274 | Tumor | 30 |
| Gene chip | GSE67268 | Normal tissue | 113 |
| Gene chip | GSE67268 | Tumor | 113 |
| High-throughput sequencing | TCGA | Normal tissue | 11 |
| High-throughput sequencing | TCGA | Tumor | 78 |
| High-throughput sequencing | GTEx | Normal tissue | 652 |
| Single-cell sequencing | GSE199619 | Tumor | 5(20793)* |
| *The number of cells included is shown in parentheses. | | | |

| Table S2. Characteristics of patients with ESCC in GSE199619 | | | | | | | | |
| --- | --- | --- | --- | --- | --- | --- | --- | --- |
| Sample ID | Age | Sex | TNM (UICC)^a^ | Stage (UICC)^a^ | Collection | NACT | Histology |  |
| ESC07 | 52 | Male | pT2N1M0 | IIIA | Surgery | none | SCC |  |
| ESC17 | 62 | Male | pT1aN2M0 | IIIA | Surgery | none | SCC |  |
| ESC18 | 74 | Female | pT1bN0M0 | IB | Surgery | none | SCC |  |
| ESC19 | 72 | Male | cT2N0M0 | IIA | Endoscopic biopsy | none | SCC |  |
| ESC21 | 69 | Male | cT4bN2M1 | IVB | Endoscopic biopsy | none | SCC |  |
| ^a^According to the Union for International Cancer Control (UICC) staging system. | | | | | | | | |
| NACT, neoadjuvant chemotherapy. | | | | | | | | |

| **Table S3. Summary of samples’information used in the experiments** | | | |
| --- | --- | --- | --- |
| Patient id | Sample id | Tissue type | Experiment type |
| hxd | hxd_t_wb | Tumor | WB |
| lgr | lgr_t_wb | Tumor | WB |
| xqt | xqt_t_wb | Tumor | WB |
| xqt | xqt_t_wb | Tumor | WB |
| hxd | hxd_n_wb | Normal | WB |
| lgr | lgr_n_wb | Normal | WB |
| xqt | xqt_n_wb | Normal | WB |
| xqt | xqt_n_wb | Normal | WB |
| hxd | hxd_N -1_section | Normal | IHC |
| hxd | hxd_N -2_section | Normal | IHC |
| hxd | hxd_T-1_section | Tumor | IHC |
| hxd | hxd_T-2_section | Tumor | IHC |
| lgr | lgr_N -1_section | Normal | IHC |
| lgr | lgr_N -2_section | Normal | IHC |
| lgr | lgr_N-3_section | Normal | IHC |
| lgr | lgr_T-1_section | Tumor | IHC |
| lgr | lgr_T-2_section | Tumor | IHC |
| lgr | lgr_T-3_section | Tumor | IHC |
| wsr | wsr_N -1_section | Normal | IHC |
| wsr | wsr_N -2_section | Normal | IHC |
| wsr | wsr_N -3_section | Normal | IHC |
| wsr | wsr_T-1_section | Tumor | IHC |
| wsr | wsr_T-2_section | Tumor | IHC |
| wsr | wsr_T-3_section | Tumor | IHC |
| xqt | xqt_N -1_section | Normal | IHC |
| xqt | xqt_N-2_section | Normal | IHC |
| xqt | xqt_N-3_section | Normal | IHC |
| xqt | xqt_T-1_section | Tumor | IHC |
| xqt | xqt_T-2_section | Tumor | IHC |
| xqt | xqt_T-3_section | Tumor | IHC |
| 42305770-10 | 42305770-10 | Tumor | Dual_IF |
| 42103977-15 | 42103977-15 | Tumor | Dual_IF |
| 42201304-12 | 42201304-12 | Tumor | Dual_IF |
| 42305256-11 | 42305256-11 | Tumor | Dual_IF |
| 42102132-16 | 42102132-16 | Tumor | Dual_IF |
| 42205599-13 | 42205599-13 | Tumor | Dual_IF |
| 42205461-7 | 42205461-7 | Tumor | Dual_IF |
| 42205472-9 | 42205472-9 | Tumor | Dual_IF |
| 72310414-1 | 72310414-1 | Tumor | Dual_IF |
| 42203022-16 | 42203022-16 | Tumor | Dual_IF |
| 42304591-20 | 42304591-20 | Tumor | Dual_IF |
| 42205446-12 | 42205446-12 | Tumor | Dual_IF |
| 42304524-11 | 42304524-11 | Tumor | Dual_IF |
| 42205728-13 | 42205728-13 | Tumor | Dual_IF |
| 42201045-11 | 42201045-11 | Tumor | Dual_IF |
| 42102022-11 | 42102022-11 | Tumor | Dual_IF |
| 42304118-12 | 42304118-12 | Tumor | Dual_IF |
| 42303764-15 | 42303764-15 | Tumor | Dual_IF |
| 42205224-5 | 42205224-5 | Tumor | Dual_IF |
| 42305203-13 | 42305203-13 | Tumor | Dual_IF |

| Table S4. Differential gene expression analysis results of core genes in the PPI network | | | | |
| --- | --- | --- | --- | --- |
| Dataset | Data type | Gene symbol | LogFC | Adj.P.Val |
| GSE161533 | Gene chip | ITGB2 | 1.319599842 | 9.63E-08 |
| GSE23400 | Gene chip | ITGB2 | 0.404887737 | 0.0000246 |
| UCSC Toil | High-throughput sequencing | ITGB2 | 1.029530039 | 1.87986E-12 |
| GSE161533 | Gene chip | CD14 | 1.763759645 | 1.79E-08 |
| GSE23400 | Gene chip | CD14 | 0.51316603 | 0.00000418 |
| UCSC Toil | High-throughput sequencing | CD14 | 1.571326582 | 1.22156E-29 |
| GSE161533 | Gene chip | CSF1R | 0.711038133 | 0.000183 |
| GSE23400 | Gene chip | CSF1R | 0.09087061 | 0.197769615 |
| UCSC Toil | High-throughput sequencing | CSF1R | 0.591320323 | 3.50765E-06 |
| GSE161533 | Gene chip | FCER1G | 2.257518197 | 6.05E-10 |
| GSE23400 | Gene chip | FCER1G | 0.631476127 | 2.95E-09 |
| UCSC Toil | High-throughput sequencing | FCER1G | 2.443424612 | 3.21673E-58 |
| GSE161533 | Gene chip | FCGR2A | 0.79959136 | 4.01E-09 |
| GSE23400 | Gene chip | FCGR2A | 0.381097149 | 4.61E-08 |
| UCSC Toil | High-throughput sequencing | FCGR2A | 2.248459042 | 1.13261E-59 |
| GSE161533 | Gene chip | ITGAM | 0.387893477 | 0.000997 |
| GSE23400 | Gene chip | ITGAM | 0.067063207 | 0.138376808 |
| UCSC Toil | High-throughput sequencing | ITGAM | 0.823767562 | 2.18249E-10 |
| GSE161533 | Gene chip | ITGAX | 0.591751969 | 1.23E-08 |
| GSE23400 | Gene chip | ITGAX | 0.102743191 | 0.009567733 |
| UCSC Toil | High-throughput sequencing | ITGAX | 0.322425118 | 0.060141412 |
| GSE161533 | Gene chip | TLR4 | 0.149992315 | 0.055635672 |
| GSE23400 | Gene chip | TLR4 | 0.079048398 | 0.012075242 |
| UCSC Toil | High-throughput sequencing | TLR4 | 0.549550655 | 1.55571E-06 |
| GSE161533 | Gene chip | C1QB | 1.836664781 | 0.000000138 |
| GSE23400 | Gene chip | C1QB | 0.824471511 | 0.000000165 |
| GSE161533 | Gene chip | TYROBP | 1.730654919 | 2.06E-08 |
| GSE23400 | Gene chip | TYROBP | 0.717503758 | 5.51E-08 |
| UCSC Toil | High-throughput sequencing | TYROBP | 2.013301476 | 1.02621E-54 |

| Table S5. Survival analysis results of core genes in the PPI network | | | | | | | |  |
| --- | --- | --- | --- | --- | --- | --- | --- | --- |
| Gene symbol | Cutpoint* | Statistic* | High (cases) | Low (cases) | Event | P value |  |  |
| CSF1R | 4.17208909 | 2.162809384 | 33 | 45 | OS | 0.014842793 |  |  |
| CSF1R | 4.17208909 | 2.783078379 | 33 | 45 | DSS | 0.002126299 |  |  |
| CSF1R | 3.841906411 | 2.593796301 | 39 | 39 | PFI | 0.007890253 |  |  |
| ITGB2 | 3.415896923 | 2.250356918 | 48 | 30 | OS | 0.026231304 |  |  |
| ITGB2 | 3.47348404 | 2.888521863 | 47 | 31 | DSS | 0.003701459 |  |  |
| ITGB2 | 3.47348404 | 2.026941702 | 47 | 31 | PFI | 0.045716685 |  |  |
| ITGAM | 0.875725154 | 2.344180811 | 64 | 14 | OS | 0.043976026 |  |  |
| ITGAM | 0.315225169 | 1.905011349 | 71 | 7 | DSS | 0.116131737 |  |  |
| ITGAM | 1.386345803 | 1.004116387 | 51 | 27 | PFI | 0.31620056 |  |  |
| TYROBP | 5.498283248 | 1.926239334 | 55 | 23 | OS | 0.072042446 |  |  |
| TYROBP | 6.868157299 | 2.651615505 | 26 | 52 | DSS | 0.001366199 |  |  |
| TYROBP | 6.002868987 | 2.139886049 | 51 | 27 | PFI | 0.038328438 |  |  |
| FCGR2A | 5.239857751 | 1.478065815 | 10 | 68 | OS | 0.059080792 |  |  |
| FCGR2A | 2.927065142 | 2.562631812 | 60 | 18 | DSS | 0.022282481 |  |  |
| FCGR2A | 4.246214791 | 1.938201238 | 33 | 45 | PFI | 0.047751346 |  |  |
| CD14 | 5.014319231 | 2.037344947 | 61 | 17 | OS | 0.058364148 |  |  |
| CD14 | 5.947827143 | 2.661612048 | 37 | 41 | DSS | 0.003705189 |  |  |
| CD14 | 6.183010271 | 2.755728653 | 28 | 50 | PFI | 0.003067691 |  |  |
| FCER1G | 5.008991173 | 2.151415387 | 69 | 9 | OS | 0.096840683 |  |  |
| FCER1G | 7.160332606 | 2.838233415 | 24 | 54 | DSS | 0.000634883 |  |  |
| FCER1G | 5.939351702 | 2.300367088 | 52 | 26 | PFI | 0.02616616 |  |  |
| ITGAX | 2.042424732 | 2.138649835 | 53 | 25 | OS | 0.038860645 |  |  |
| ITGAX | 2.281018784 | 2.426981372 | 46 | 32 | DSS | 0.016616476 |  |  |
| ITGAX | 2.819767212 | 1.883825425 | 29 | 49 | PFI | 0.045580838 |  |  |
| TLR4 | 0.371359287 | 2.006880727 | 65 | 13 | OS | 0.079432499 |  |  |
| TLR4 | 0.303811262 | 2.10772992 | 67 | 11 | DSS | 0.08795124 |  |  |
| TLR4 | 0.319029821 | 2.71579259 | 66 | 12 | PFI | 0.018745618 |  |  |
| C1QB | 5.677135957 | 2.637116599 | 64 | 14 | OS | 0.027386055 |  |  |
| C1QB | 7.50172583 | 2.994255858 | 32 | 46 | DSS | 0.00087659 |  |  |
| C1QB | 5.677135957 | 2.754899435 | 64 | 14 | PFI | 0.015142551 |  |  |
| *The optimal cut-off points and their statistical values obtained by R package "survminer". | | | | | | | | |

| Table S6. The expression of ITGB2 in macrophages compared to other cells | | | | | | | | |
| --- | --- | --- | --- | --- | --- | --- | --- | --- |
| Cell_1 | Cell_2 | Gene_symbol | p_val | avg_log2FC | pct.1 | pct.2 | p_val_adj |  |
| Macrophages | B cells | ITGB2 | 5.4259E-172 | 0.706781289 | 0.559 | 0.286 | 1.6243E-167 |  |
| Macrophages | Endothelium | ITGB2 | 3.28999E-40 | 1.038403671 | 0.559 | 0.032 | 9.84892E-36 |  |
| Macrophages | Epithelium | ITGB2 | 1.083E-252 | 1.003815767 | 0.559 | 0.038 | 3.2421E-248 |  |
| Macrophages | Fibroblast | ITGB2 | 1.1972E-233 | 0.819099538 | 0.559 | 0.107 | 3.5838E-229 |  |
| Macrophages | Mast cells | ITGB2 | 3.05392E-36 | 0.779055829 | 0.559 | 0.202 | 9.1422E-32 |  |
| Macrophages | Other myeloid cells | ITGB2 | 1.62935E-18 | 0.241952739 | 0.559 | 0.455 | 4.87763E-14 |  |
| Macrophages | T+NK cells | ITGB2 | 1.5699E-149 | 0.402515482 | 0.559 | 0.407 | 4.6997E-145 |  |

| **Table S7. M2 polarization signature gene set** |
| --- |
| M2 polarization |
| ARG1 |
| ARG2 |
| IL10 |
| CD32 |
| CD163 |
| CD23 |
| CD200R1 |
| PDCD1LG2 |
| CD274 |
| MARCO |
| CSF1R |
| CD206 |
| IL1RN |
| IL1R2 |
| IL4R |
| CCL4 |
| CCL13 |
| CCL20 |
| CCL17 |
| CCL18 |
| CCL22 |
| CCL24 |
| LYVE1 |
| VEGFA |
| VEGFB |
| VEGFC |
| VEGFD |
| EGF |
| CTSA |
| CTSB |
| CSTC |
| CTSD |
| TGFB1 |
| TGFB2 |
| TGFB3 |
| MMP14 |
| MMP19 |
| MMP9 |
| CLEC7A |
| WNT7B |
| FASL |
| TNFSF12 |
| TNFSF8 |
| CD276 |
| VTCN1 |
| MSR1 |
| FN1 |
| IRF4 |

| **Table S8. Correlation between ITGB2 and M2 Macrophage markers** | | | | | |
| --- | --- | --- | --- | --- | --- |
| Dataset | Gene | Marker | Cor | P_value |  |
| GSE161533 | ITGB2 | CD163 | 0.686599 | P<0.0001 |  |
| GSE161533 | ITGB2 | MRC1 | 0.431856 | P=0.0227 |  |
| GSE23400 | ITGB2 | CD163 | 0.732704 | P<0.0001 |  |
| GSE23400 | ITGB2 | MRC1 | 0.752459 | P<0.0001 |  |
| TCGA | ITGB2 | CD163 | 0.779208 | P<0.0001 |  |
| TCGA | ITGB2 | MRC1 | 0.646303 | P<0.0001 |  |

| **Table S9. The origin of the 16 TME gene sets related to immunotherapy response** | | | |  |
| --- | --- | --- | --- | --- |
| Signatures | Published year | Journal | PMID | |
| CD_8_T_effector | 2018 | Nature | 29443960 | |
| APM | 2018 | nature | 29443960 | |
| CD8_Rooney_et_al | 2015 | Cell | 25594174 | |
| pDCs_Rooney_et_al | 2015 | Cell | 25594174 | |
| Co_stimulation_APC  _Rooney_et_al | 2015 | Cell | 25594174 | |
| Co_stimulation_T  _cell_Rooney_et_al | 2015 | Cell | 25594174 | |
| Type_I_IFN_Reponse  _Rooney_et_al | 2015 | Cell | 25594174 | |
| T_cell_inflamed_GEP  _Ayers_et_al | 2017 | The Journal of Clinical Investigation | 28650338 | |
| IFNG_signature  _Ayers_et_al | 2017 | The Journal of Clinical Investigation | 28650338 | |
| MHC_Class_II | 2020 | IOBR | - | |
| MHC_Class_I | 2020 | IOBR | - | |
| Antigen_Processing_and  _Presentation_Li_et_al | 2020 | Nature Communication | 32081859 | |
| Chemokines_Li_et_al | 2020 | Nature Communication | 32081859 | |
| Chemokine_Receptors  _Li_et_al | 2020 | Nature Communication | 32081859 | |
| TIP_Priming_and  _activation_1 | 2018 | Cancer Research | 30154154 | |
| TIP_Priming_and  _activation_2 | 2018 | Cancer Research | 30154154 | |

| **Table S10. The Correlation of ITGB2 with 16 TME Features Related to Immunotherapy Response** | | | | |
| --- | --- | --- | --- | --- |
| Dataset | gene | TME | Cor | P_value |
| GSE23400 | ITGB2 | CD_8_T_effector | 0.645541042 | 4.04455E-07 |
| GSE23400 | ITGB2 | APM | 0.43718755 | 0.001180901 |
| GSE23400 | ITGB2 | CD8_Rooney_et_al | 0.564183196 | 1.55041E-05 |
| GSE23400 | ITGB2 | pDCs_Rooney_et_al | 0.566440897 | 1.41421E-05 |
| GSE23400 | ITGB2 | Co_stimulation_APC_Rooney_et_al | 0.358168037 | 0.008785386 |
| GSE23400 | ITGB2 | Co_stimulation_T_cell_Rooney_et_al | 0.478551846 | 0.000340414 |
| GSE23400 | ITGB2 | Type_I_IFN_Reponse_Rooney_et_al | 0.38034188 | 0.005232076 |
| GSE23400 | ITGB2 | MHC_Class_I | 0.504596033 | 0.000143858 |
| GSE23400 | ITGB2 | MHC_Class_II | 0.786002258 | 0 |
| GSE23400 | ITGB2 | T_cell_inflamed_GEP_Ayers_et_al | 0.687631027 | 4.72133E-08 |
| GSE23400 | ITGB2 | IFNG_signature_Ayers_et_al | 0.633607483 | 7.16661E-07 |
| GSE23400 | ITGB2 | TIP_Priming_and_activation_1 | 0.627560071 | 9.5387E-07 |
| GSE23400 | ITGB2 | TIP_Priming_and_activation_2 | 0.635945815 | 6.41181E-07 |
| GSE23400 | ITGB2 | Antigen_Processing_and_Presentation_Li_et_al | 0.701902919 | 1.91756E-08 |
| GSE23400 | ITGB2 | Chemokines_Li_et_al | 0.681825512 | 6.50955E-08 |
| GSE23400 | ITGB2 | Chemokine_Receptors_Li_et_al | 0.599500081 | 3.44952E-06 |
| TCGA | ITGB2 | CD_8_T_effector | 0.589220906 | 2.67984E-08 |
| TCGA | ITGB2 | APM | 0.54109182 | 4.99127E-07 |
| TCGA | ITGB2 | CD8_Rooney_et_al | 0.464940123 | 2.24725E-05 |
| TCGA | ITGB2 | pDCs_Rooney_et_al | 0.470175394 | 1.77444E-05 |
| TCGA | ITGB2 | Co_stimulation_APC_Rooney_et_al | 0.312788477 | 0.00548019 |
| TCGA | ITGB2 | Co_stimulation_T_cell_Rooney_et_al | 0.535628928 | 6.74668E-07 |
| TCGA | ITGB2 | Type_I_IFN_Reponse_Rooney_et_al | 0.29091162 | 0.009992982 |
| TCGA | ITGB2 | MHC_Class_I | 0.547920434 | 3.40099E-07 |
| TCGA | ITGB2 | MHC_Class_II | 0.739071056 | 0 |
| TCGA | ITGB2 | T_cell_inflamed_GEP_Ayers_et_al | 0.584769661 | 3.61832E-08 |
| TCGA | ITGB2 | IFNG_signature_Ayers_et_al | 0.622655825 | 1.06949E-09 |
| TCGA | ITGB2 | TIP_Priming_and_activation_1 | 0.55783457 | 1.92013E-07 |
| TCGA | ITGB2 | TIP_Priming_and_activation_2 | 0.716814831 | 0 |
| TCGA | ITGB2 | Antigen_Processing_and_Presentation_Li_et_al | 0.746506658 | 0 |
| TCGA | ITGB2 | Chemokines_Li_et_al | 0.555785986 | 2.16416E-07 |
| TCGA | ITGB2 | Chemokine_Receptors_Li_et_al | 0.608062823 | 6.31668E-09 |

| **Table S11. Clinical trials relevant to the selected macrophage targets in this study** | | | |  |
| --- | --- | --- | --- | --- |
| Compound (sponsor) | Clinical phase (status) | Tumour type | NCT identifier | |
| Carlumab (anti-CCL2  antibodies; Centocor) | Phase II (completed) | Prostate cancer | NCT00992186 | |
| BMS-813160 (CCR2/CCR5  antagonist; Bristol Myers Squibb) | Phase II (ongoing) | Renal carcinoma | NCT02996110 | |
| BMS-813160 (CCR2/CCR5  antagonist; Bristol Myers Squibb) | Phase I/II (ongoing) | Pancreatic cancer, CRC, NSCLC | NCT03184870 | |
| BMS-813160 (CCR2/CCR5  antagonist; Bristol Myers Squibb) | Phase II (ongoing) | Hepatocellular carcinoma | NCT04123379 | |
| CCR5 antagonist (Pfizer) | Phase I (completed) | CRC | NCT03274804 | |
| CCR6 antagonist (Pfizer) | Phase I (ongoing) | Phase I (ongoing) | NCT04721301 | |
| PLX3397 (Plexxikon;  CSF1R inhibitors) | Phase I/II (ongoing) | Sarcoma, nerve-sheath tumours | NCT02584647 | |
| PLX3397 (Plexxikon;  CSF2R inhibitors) | Phase I/II (ongoing) | Advanced melanoma and solid tumours | NCT02452424 | |
| PLX3397 (Plexxikon;  CSF3R inhibitors) | Phase I/II (ongoing) | Breast cancer | NCT01596751 | |
| LY3022855 (IMC-CS4; Eli  Lilly;Anti-CSF1R antibodies) | Phase I/II (ongoing) | Melanoma | NCT03101254 | |
| Hu5F9-G4 (Stanford University;  Anti-CD47/SIRPα antibodies) | Phase I/II (completed) | Solid tumours | NCT02953782 | |
| Hu5F9-G4 (Stanford University;  Anti-CD48/SIRPα antibodies) | Phase I/II (ongoing) | Urothelial and bladder cancer | NCT03869190 | |
| ALX148 (ALX Oncology;  CD47–Fc fusion protein) | Phase II (ongoing) | HNSCC | NCT04675294 | |
| ALX148 (ALX Oncology;  CD48–Fc fusion protein) | Phase II (ongoing) | HNSCC | NCT04675333 | |
| SEA-CD40 (Seagen) | Phase I (ongoing) | Solid and haematological tumours | NCT02376699 | |
| (Nanoplexed TLR3 agonist; Highlight Therapeutics;TLR3 agonists) | Phase II (ongoing) | Melanoma | NCT04570332 | |
| BDC-1001 (TLR7/8 agonist plus  trastuzumab Bolt Biother) | Phase I/II (ongoing) | HER2+ solid tumours | NCT04278144 | |
| PY314 (anti-TREM2 antibodies;  Pionyr Immunotherapeutics) | Phase I (ongoing) | Solid tumours | NCT04691375 | |
